# Searching glycolate oxidase inhibitors based on QSAR, molecular docking, and molecular dynamic simulation approaches

**DOI:** 10.1038/s41598-022-24196-4

**Published:** 2022-11-19

**Authors:** Nicolás Cabrera, Sebastián A. Cuesta, José R. Mora, José Luis Paz, Edgar A. Márquez, Patricio J. Espinoza-Montero, Yovani Marrero-Ponce, Noel Pérez, Ernesto Contreras-Torres

**Affiliations:** 1 Department of Biomedical Engineering, Texas A&M University, College Station, TX 77843 USA; 2 Department of Chemistry, Manchester Institute of Biotechnology, The University of Manchester, 131 Princess Street, Manchester, M1 7DN UK; 3 Instituto de Simulación Computacional (ISC-USFQ), Departamento de Ingeniería Química, Universidad San Francisco de Quito, Diego de Robles y Vía Interoceánica, 170901 Quito, Ecuador; 4 Departamento Académico de Química Inorgánica, Facultad de Química e Ingeniería Química, Universidad Nacional Mayor de San Marcos, Lima, Perú; 5 Grupo de Investigaciones en Química y Biología, Departamento de Química y Biología, Facultad de Ciencias Básicas, Universidad del Norte, Carrera 51B, Km 5, Vía Puerto Colombia, 081007 Barranquilla, Colombia; 6 Escuela de Ciencias Químicas, Pontificia Universidad Católica del Ecuador, 17012184 Quito, Ecuador; 7 Grupo de Medicina Molecular y Traslacional (MeM&T), Escuela de Medicina, Colegio de Ciencias de la Salud (COCSA), Universidad San Francisco de Quito (USFQ), Edificio de Especialidades Médicas, Diego de Robles y Vía Interoceánica, 170157 Quito, Pichincha Ecuador; 8 Departamento de Ciencias de la Computación, Centro de Investigación Científica y de Educación Superior de Ensenada (CICESE), Ensenada, México; 9 Colegio de Ciencias e Ingenierías “El Politécnico”, Universidad San Francisco de Quito (USFQ), 170901 Quito, Ecuador; 10 CAM – Basque Center for Applied Mathematics, Mazarredo 14, 48009 Bilbao, Basque Country, Spain

**Keywords:** Computational biology and bioinformatics, Drug discovery

## Abstract

Primary hyperoxaluria type 1 (PHT1) treatment is mainly focused on inhibiting the enzyme glycolate oxidase, which plays a pivotal role in the production of glyoxylate, which undergoes oxidation to produce oxalate. When the renal secretion capacity exceeds, calcium oxalate forms stones that accumulate in the kidneys. In this respect, detailed QSAR analysis, molecular docking, and dynamics simulations of a series of inhibitors containing glycolic, glyoxylic, and salicylic acid groups have been performed employing different regression machine learning techniques. Three robust models with less than 9 descriptors—based on a tenfold cross (Q^2^_CV_) and external (Q^2^_EXT_) validation—were found i.e., MLR1 (Q^2^_CV_ = 0.893, Q^2^_EXT_ = 0.897), RF1 (Q^2^_CV_ = 0.889, Q^2^_EXT_ = 0.907), and IBK1 (Q^2^_CV_ = 0.891, Q^2^_EXT_ = 0.907). An ensemble model was built by averaging the predicted pIC_50_ of the three models, obtaining a Q^2^_EXT_ = 0.933. Physicochemical properties such as charge, electronegativity, hardness, softness, van der Waals volume, and polarizability were considered as attributes to build the models. To get more insight into the potential biological activity of the compouds studied herein, docking and dynamic analysis were carried out, finding the hydrophobic and polar residues show important interactions with the ligands. A screening of the DrugBank database V.5.1.7 was performed, leading to the proposal of seven commercial drugs within the applicability domain of the models, that can be suggested as possible PHT1 treatment.

## Introduction

**Figure 1:**
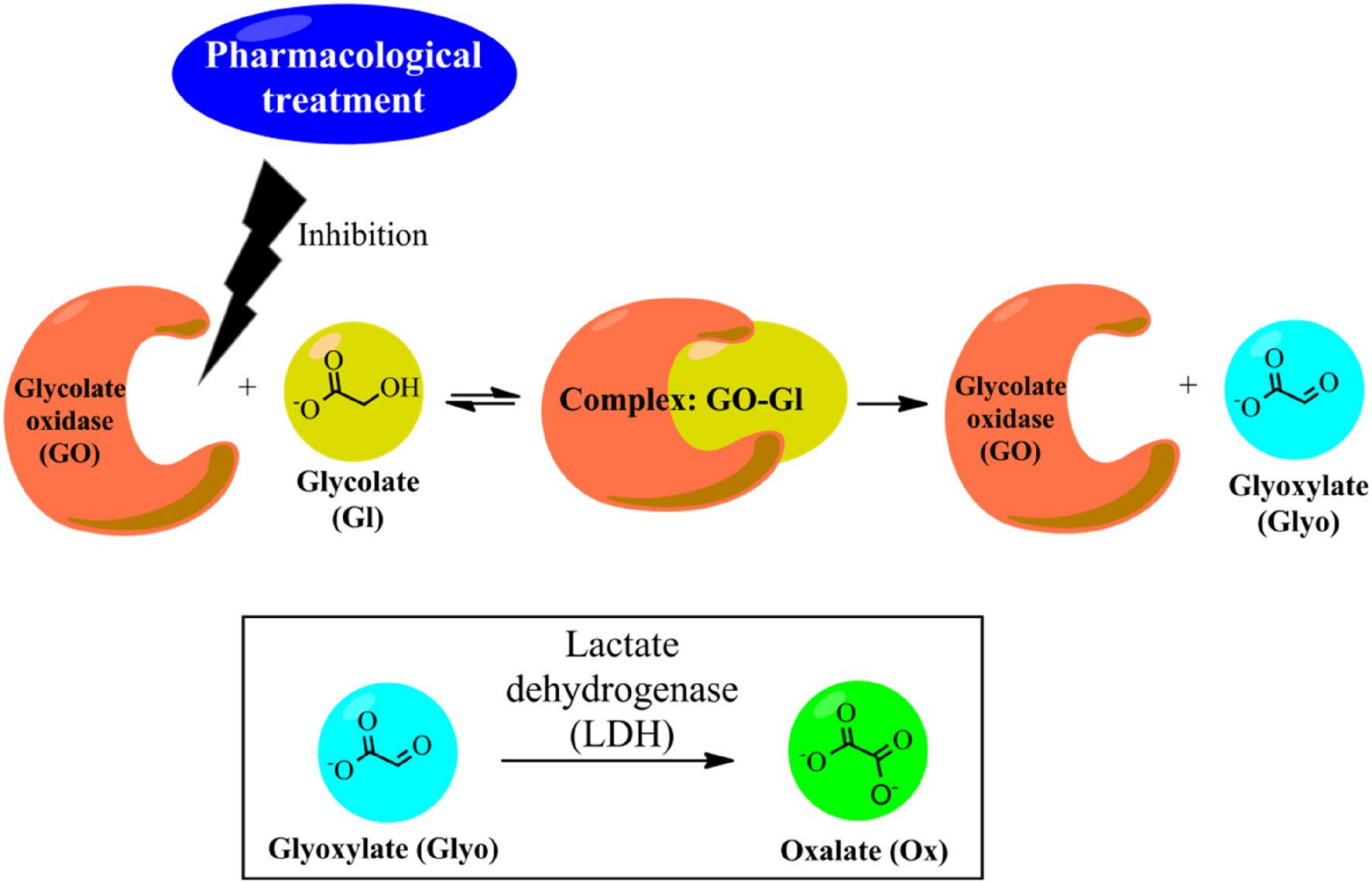
Schematic representation of the production of oxalate from glycolate. Detail flow: A pharmacological treatment that inhibit GO and GI interaction may reduce the production of Ox that forms insoluble kidney stones.

Primary hyperoxaluria type 1 (PHT1) is a genetic alteration of the hepatic glycolate (Gl) metabolism^1^ that can be treated by inhibiting the glycolate oxidase (GO). PHT1 is caused by the deficiency of the liver peroxisomal enzyme glyoxylate aminotransferase^2^ and is mainly treated by a double kidney-liver transplant, although the survival rates are low^3,4^. GO has been targeted for the design of possible pharmacological treatments for PHT1. Several candidates for GO inhibition have been studied with different results, mainly focused on the presence of a carboxylic acid group and analogues in the structure to mimic the glycolate substrate. In this sense, GO inhibition reduces the production of glyoxylate (Glyo)^5^. First, Glyo is produced from the oxidation of glycolate (Gl) by the action of the GO enzyme^5–8^. Then, the lactate dehydrogenase (LDH) overproduces oxalate (Ox) forming insoluble crystals of calcium oxalate in the kidney and other tissues^9^. A schematic representation of the oxidation process from Gl to Ox is depicted in Fig. 1**.**

The potency of possible GO inhibitors was proved in vitro and determined the half-maximal inhibitory concentration (IC_50_). In this regard, in 1979, Randall et al*.*^10^ explored the biological activity of three possible GO inhibitors, i.e., substituted glycolic acids, oxyacetic acids, and glyoxylic acids. In 1983, Rooney et al*.*^11^and Williams et al*.*^12^ explored a series of inhibitors with acidic groups in their structure, demonstrating that lipophilicity is essential in the catalytic activity. In 2018, Moya-Garzón et al*.*^13^, explored a series of salicylic acid derivatives. Furthermore, their results suggested that a hydrophobic tail and electron-rich heterocycles increase the performance of GO inhibitors, describing the importance of a linker for the correct accommodation of the molecule in the enzyme for this kind of derivatives.

Quantitative Structure–Activity Relationship (QSAR), molecular docking, and molecular dynamics can give valuable information about the inhibition process. QSAR analysis is an efficient technique to understand how the chemical structure of a compound is related to its biological activity^14^. Moreover, QSAR has been applied for different clinical diseases like diabetes^15^, human immunodeficiency virus (HIV)^16^, Alzheimer^17^, cancer^1^^18–20^, and malaria^21,22^. Molecular docking and molecular dynamics are atomistic simulations that underline protein–ligand binding—key to achieving inhibition^23–25^. With these methods, the ligand-receptor interaction can be explored, and the formation of stable complexes predicted. For example, molecular docking is a powerful technique used to predict the interaction between small drugs and glyoxylate aminotransferase (AGT), identifying promising compounds for PHT1^26^.

This study used molecular dynamics to obtain more insights into the interactions aiming for a novel PHT1 treatment. Molecular dynamics play a pivotal role in evaluating the complex's stability over time, considering essential conditions such as the solvent effect, salt concentration temperature, pressure, volume, and the number of particles^27^. These methods are recommended to explain the relationship between computational and experimental findings^28^.

Due to the need for a novel PHT1 treatment, this study presents QSAR individual and ensemble models to predict the inhibitory potency of GO inhibitors. To date, QSAR analysis has not been used to explore PHT1 treatments. Therefore, the dataset of 144 GO inhibitors was collected from the literature^10–13^ and used for the QSAR analysis by employing topographic descriptors^29^. These descriptors were obtained from the chemical structure of the candidates based on internal atomic arrangement, molecule size, shape, branching, and the presence of heteroatoms or multiple bonds^30^. Furthermore, a deeper analysis was performed with docking and molecular dynamics analysis on the GO active sites obtained from the crystallographic data^31^. In addition, a screening of the DrugBank database was performed, and a list of commercial drugs was suggested for PHT1 treatment by targeting the enzyme glycolate oxidase. In addition, the screening over DrugBank gives us a unique advantage: these compounds are already proven, and well-studied drugs; therefore, the possibility to be used for new treatments could be shorter.

## Results and discussion

### Dataset, descriptors, and QSAR modelling

Nine out of forty subsets—composed of less than 13 topographical descriptors—were individual models. All the models meet the criteria of high ${Q}_{CV}^{2}$, and low MAE when they are trained to predict the pIC_50_ values (Table S2, Fig. S1). The individual models with the highest ${Q}_{CV}^{2}$ and the smallest MAE_CV_ of each technique were labelled as IBK1, MLR1, and RF1.

**Figure 2:**
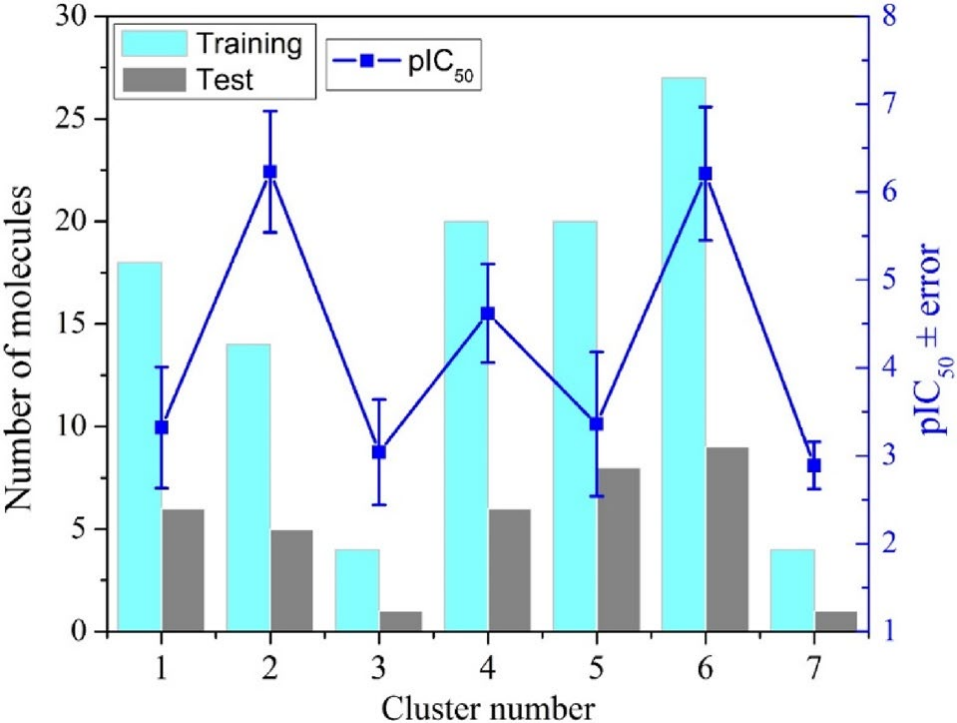
Training and test set splitting employing the K-means technique (25% test and 75% training).

The dataset was separated into seven clusters (Fig. 2) based on Ward´s method, which minimize the error of the sum of squares within clusters. 25% of the molecules were labelled as test sets and 75% as the training set (Fig. 2). Each cluster contains between 5 and 36 molecules. In each cluster, the molecules were ordered from smaller to bigger pIC_50_ values, and 25% were randomly selected to form part of the test set. The rest were kept in the training set.

The applicability domain (AD) was applied using the training sets of the models IBK1, MLR1, and RF1 using the consensus approach as implemented in AMBIT. The AD analysis was applied to the test set on each model where only compound **84** was considered outside of the AD for the IBK1 and MLR1 models, suggesting a comprehensive coverage, with more than 97% of molecules being within the AD. Consequently, compound **84** was removed, and the statistical parameters were calculated on the training and test (Table 1 and S2).

**Table 1:** R^2^_ADJ_, Q^2^_EXT_, Q^2^_CV_, number of features, and MAE obtained with MLR1, IBK1, and RF1.

Model ID	Number of features	R^2^_ADJ_	MAE	Q^2^_CV_	MAE_CV_	Q^2^_EXT_	MAE_EXT_
IBK1	7	0.942	0.270	0.891	0.413	0.907	0.364
MLR1	8	0.915	0.337	0.893	0.383	0.897	0.376
RF1	7	0.987	0.134	0.889	0.382	0.907	0.310

**Figure 3:**
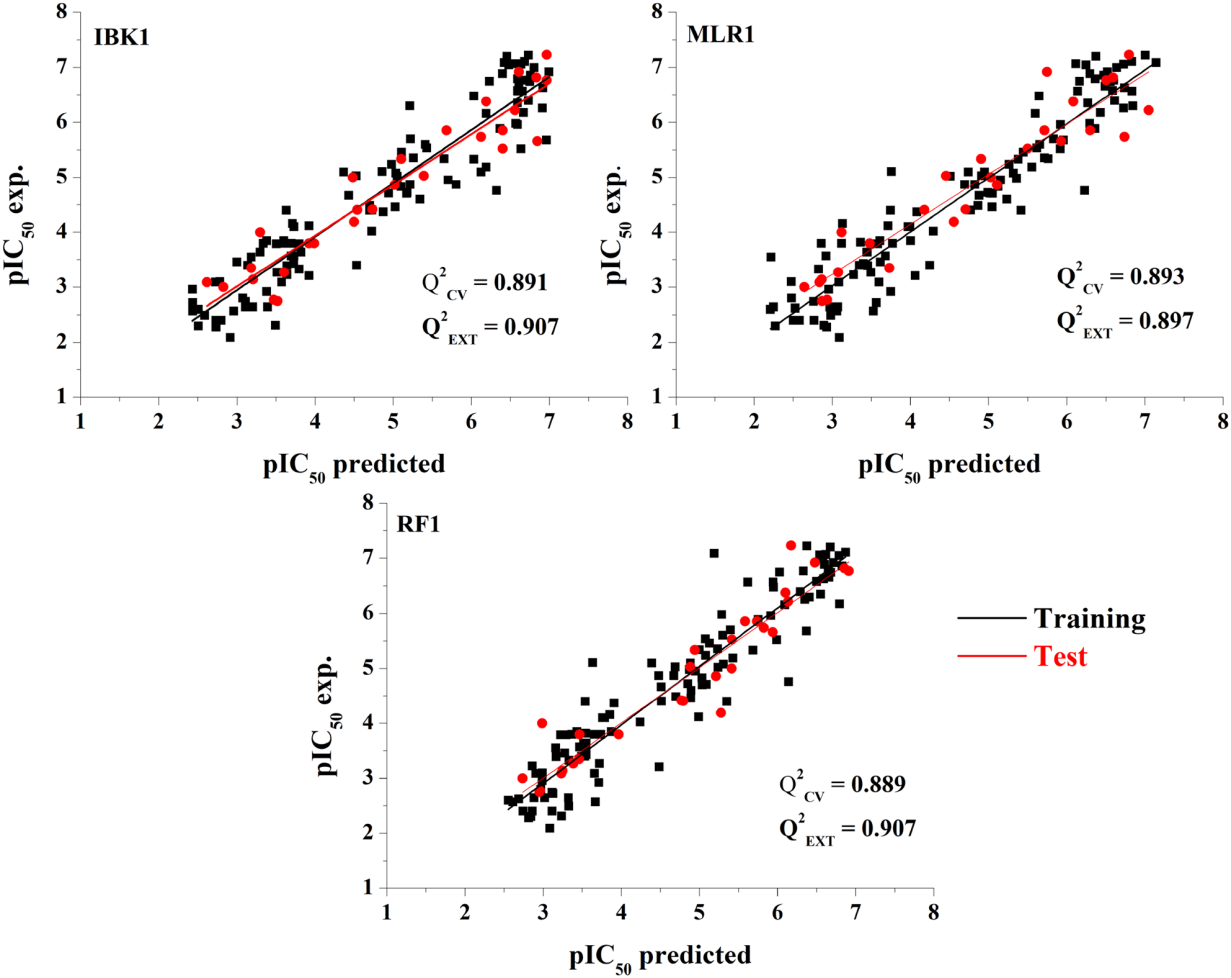
Experimental versus predicted pIC_50_ values obtained by models MLR1, RF1 and IBK1.

Figure 3 presents the plot of the predicted vs experimental values of the pIC_50_ for MLR1, IBK1, and RF1, considering the prediction of the external test set validation coefficient (Q^2^_EXT_), and the prediction from the tenfold cross-validation (Q^2^_CV_). Q^2^_EXT_ values greater than 0.89 suggest that these models are robust enough for the prediction of the GO inhibitory activity of this dataset. A good fitting is also observed for the three models when experimental vs calculated pIC_50_ is plotted (R^2^ > 0.9, Table 1). The descriptors found for these three models are presented in the supplementary information and their description below (Table S3).

Physicochemical properties charge (c), electronegativity (e), hardness (h), softness (s), Van der Waals volume (v), and polarizability (p) were found as descriptors The realfull name of each feature is available in the supplementary materials (Table S3). These topographical descriptors have demonstrated a good capacity for building models to predict pIC_50_ values in different diseases^32–36^.

Considering that the charge distribution in the molecules can be related to their polarity, the presence of charge can be attributed to the polar functional groups present in the dataset, such as carboxylic acid, alcohols, and amine groups, among others. This result agrees with the previous results reported in the literature^10–13^. The charge distribution is also related to the existence of charge transfer complexes in the drug interaction with the active site in the enzymes^37^. Based on the presence of electronegative atoms in the molecular core, such as O, N, and S, the electronegativity index plays a pivotal role in drug design which can be helpful in the formation of electrostatic interactions and the formation of hydrogen bonds^38–40^. The hardness and softness properties give information about the HOMO–LUMO interactions, which represent the changes in polarization (Polarizability index) on the molecules due to the presence of small perturbation; this kind of polarization changes is related to the energy gap HOMO–LUMO^22,41,42^. The van der Waals volume is a physical property widely employed in QSAR modelling. It is essential in topographic attributes taking into account the information of molecular size and its possible incorporation into the active site pocket^43–45^.

**Figure 4:**
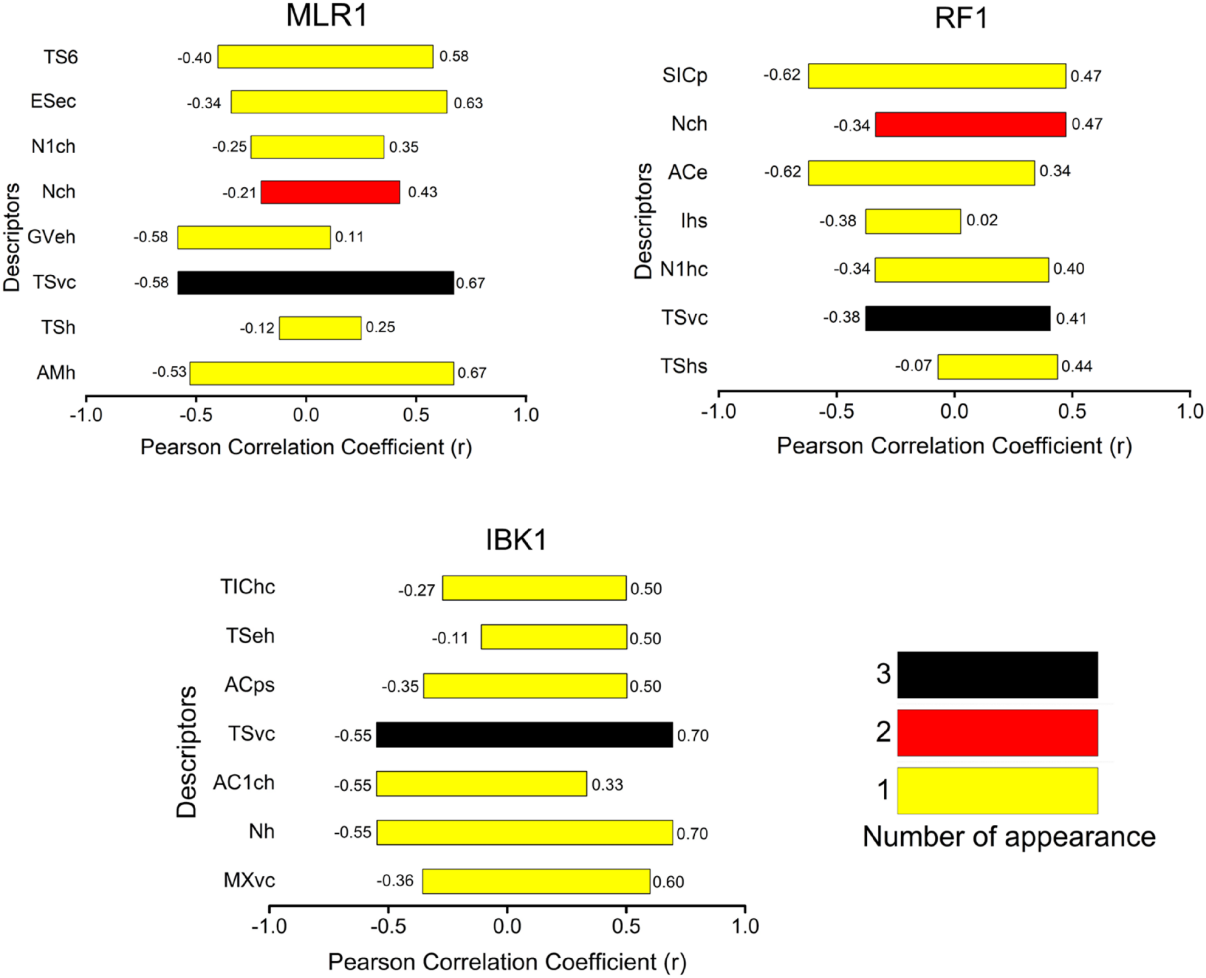
Graphical representation of the maximum and minimum values of Pearson correlation coefficients (r) for each descriptor vs the rest in MLR1, RF1 and IBK1 models. Values for the corresponding correlation matrix are presented in Tables S4, S5 and S6.

It is important to highlight that relevant criteria for the validation of the model are the ones related to the analysis of the collinearity between descriptors. A robust model requires a small Pearson coefficient (r < 0.7) between descriptors. This avoids redundant information and overfitting in the model. Collinearity between the descriptors of the training set was evaluated by using the Pearson coefficient (r), as implemented in the software^46^ (https://dunant.dista.uninsubria.it/qsar/?page_id=37), and the corresponding correlation matrix of each one is presented in Tables S4, S5 and S6. The correlation between descriptors was presented graphically using these coefficients' maximum and minimum values (Fig. 4).

Furthermore, the frequency of each descriptor in the different models was assessed. As observed in the histograms, no collinearity was obtained between the descriptors, where absolute values of Pearson coefficient < 0.7 were obtained. Based on the number of appearances of each descriptor in the models (Fig. 4), suggest a diversity of information within the models required for constructing an ensemble. TSvc is the only descriptor in common.

### Ensemble model

**Figure 5:**
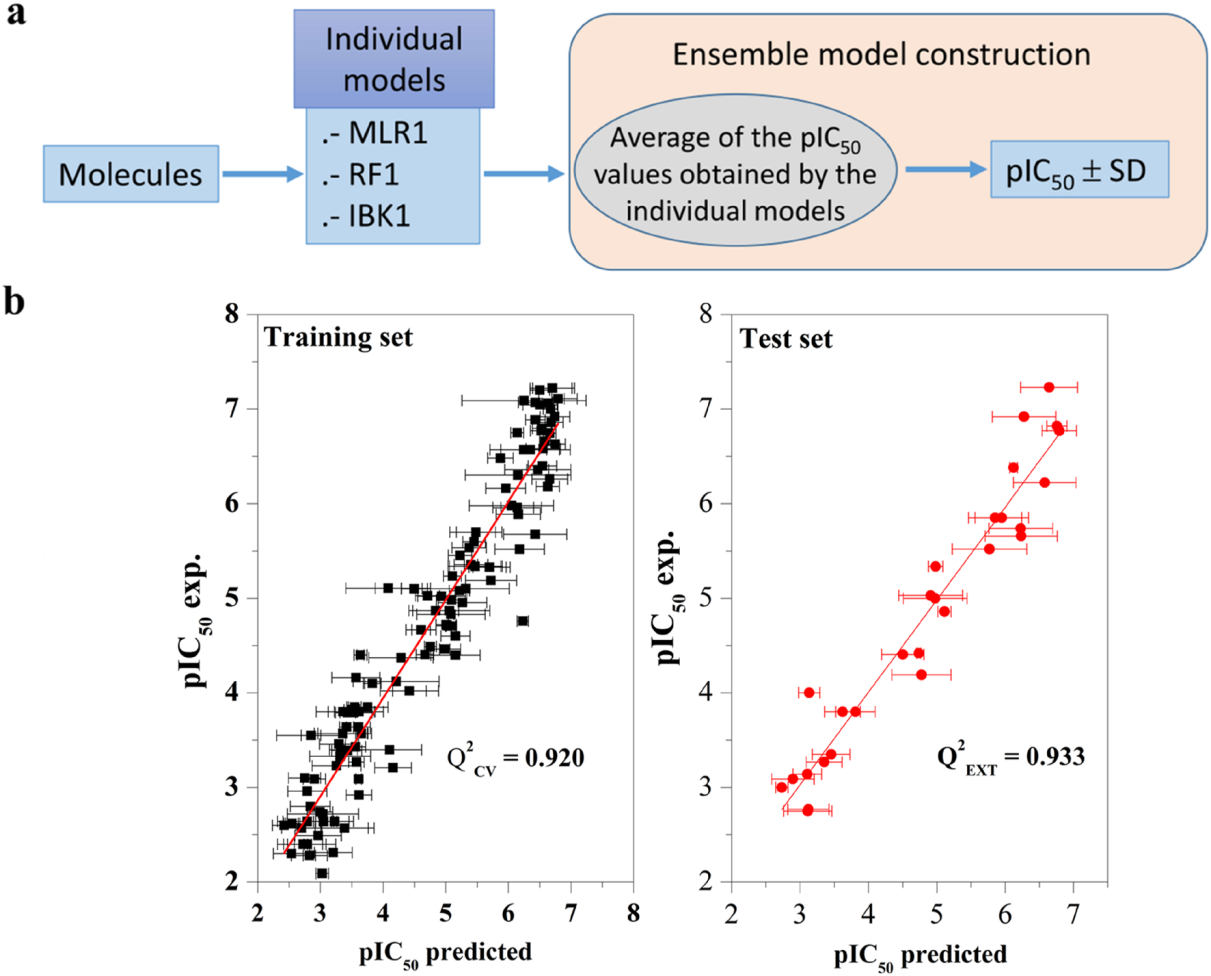
(**a**) Schematic representation for the ensemble construction procedure. (**b**) pIC_50_ experimental vs. pIC_50_ predicted the training and test sets obtained with the ensemble model.

An ensemble model is a combination of individual models to enhance its prediction. The effectiveness of the ensemble model is highly dependent on the independence of the inaccuracy committed by the individual models. In this respect, ensemble model was constructed, , with the average of the predicted pIC_50_ values of the three robust models (MLR1, RF1, and IBK1 (Fig. 5a); The predicted values obtained with the ensemble model were plotted versus the experimental values of the training and test sets (Fig. 5b), suggesting a excellent linear correlation between predicted and experimental values with a Q^2^_CV_ of 0.920 and a Q^2^_EXT_ of 0.933. The bar error of each prediction is included based on the standard deviation of the average value of each predicted pIC_50_ value.

### Drug bank screening

A screening of the DrugBank database was performed to evaluate the possibility of finding approved, experimental, and/or withdrawn drugs which can have potential application in the PHT1 treatment as an alternative use of the drug. The ensemble model obtained was used to predict the pIC_50_ of the compounds from this database.

Considering the applicability domain on the DrugBank database for the models MLR1, IBK1, and RF1, it was found that 4239, 2960, and 6670 compounds meet the requirements to be within the AD of each model, respectively. Then, for calculating the pIC_50_ through the ensemble model, 2479 compounds were considered, within the applicability domain of three individual models. After that, compounds with predicted pIC_50_ values higher than 6.5 were filtered out ending with 130 molecules. Finally, to suggest known drugs with a possible novel GO inhibitory activity, 15 molecules (a representative sample of 12% of molecules) were chosen randomly for subsequent analysis.

Taking a closer look at these drugs, eight were taken out from the list due to their antibiotic and antiviral activity i.e. cefalotin, cefotaxime, cefapirin, 4-Methyl-N-[5-(5-methyl-furan-2-ylmethylene)-4-oxo-thiazolidin-2-ylidene]-benzenesulfonamide, Latrunculin B, (5Z)-5-[(5-ethylfuran-2-yl)methylidene]-2-[[(S)-(4-fluorophenyl)-(2H-tetrazol-5-yl)methyl]amino]-1,3-thiazol-4-one,sitafloxacin, and gemifloxacin^47–49^. With antimicrobial resistance being one of the biggest health problems in the world^50^, and the emerging antiviral resistance^51^, antibiotics and antivirals should only be used to serve the purposes they were designed for and under medical surveillance. Therefore, they are unsuitable for PHT1 treatment even if these compounds present higher inhibitory activity.

The remaining seven molecules were selected to get insights in the interactions with GO i.e. Olmesartan (pIC_50pred._ = 6.52), a member of the sartans family and an angiotensin II receptor antagonists^52,53^; benzodiazepine clorazepate (pIC_50pred._ = 6.72) which present sedative, anticonvulsant, hypnotic, sedative, and relaxant properties^54,55^; PDE_5_ inhibitor udenafil (pIC_50pred._ = 6.50) used to treat erectile dysfunction^56,57^; corticoid mometasone furoate (pIC_50pred._ = 7.10) used in allergic rhinitis, asthma, pruritus, and others^58^; 5-(2-Ethoxyethyl)-5-[4-(4-fluorophenoxy)phenoxy]pyrimidine-2,4,6(1H,3H,5H)-trione (5,5PT) (pIC_50pred._ = 6.79), an experimental drug meant to treat neuroinflammatory diseases, arthritis, and tumor invasion^59^; nematocide cambendazole (pIC_50pred._ = 6.96) used to treat worm infections mainly in cattle, horses, and sheep^60^; and floctafenine (pIC_50pred._ = 6.89), an anti-inflammatory and non-narcotic analgesic drug^61^. These seven compounds are analyzed in detail in the further molecular docking and dynamic sections.

### Molecular docking

For this analysis, the enzyme 2RDopt was built by using the X-ray diffraction crystal structure of human glycolate oxidase in a complex with glyoxylate (PDB ID: 2RDU) and with 4-carboxy-5-dodecylsulfanyl-1,2,3-triazole (CDST) (PDB ID: 2RDT). During the receptor preparation, 2RDT was aligned to 2RDU, finding a small Root Mean Square (RMS) value of 0.209, meaning both structures belong to the same enzyme (Fig. S2a), where only a missing random coil (RC) was found at the entrance of the active site in 2RDT (amino acids Lys176 to Leu205). Analyzing both crystal structures, the missing amino acids in 2RDT are attributed to the presence of CDST in the active site of GO. In 2RDU, due to the small size of Gl, RC interacts with GO reducing its vibration and making it possible to be elucidated using X-ray crystallography (Fig. S2b). On the other side, as the CDST structure presents a long tail, RC may undergo greater fluctuations, challenging to capture using this experimental technique. To have a complete GO structure for this study, 2RDopt was created as described in the methodology section removing the clashes found between CDST and the RC (Fig. S2c). An RMSD value of 0.316 was obtained between the experimental and optimized structure (Fig. S2d), which implies that only subtle changes were made to the structure during the optimization.

**Figure 6:**
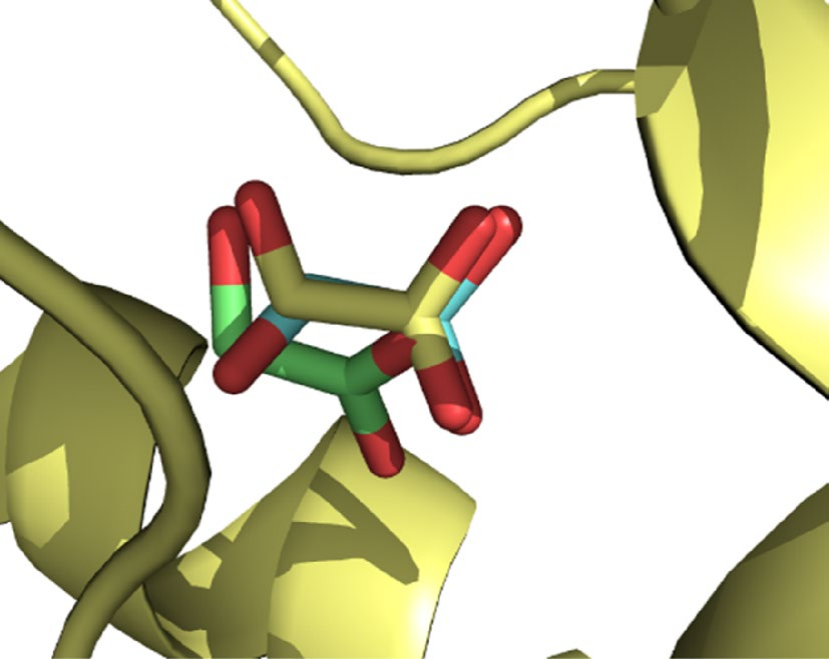
Glyoxylate conformation comparison between experimental (*yellow*), 2RDT docked (*green*), and 2RDopt (*turquoise*).

To determine the importance of the random coil, the 144 structures involved in the study were docked against 2RDT and 2RDopt. However, first, the results were validated by comparing Gl's experimental and docked conformations inside the GO active site (Fig. 6).

Figure 6 shows that the experimental pose of Gl overlays the ones obtained computationally, showing a better fit in 2RDopt docked structures. This proves the feasibility of docking calculations as a tool to predict the binding mode of GO inhibitors. Comparing both results, docking scores generally present more negative values for 2RDopt than for 2RDT, suggesting the RC may be essential for target-ligand interaction. Docking scores for 2RDT range from − 8.8 kcal/mol to − 3.3 kcal/mol, while those for 2RDopt from − 12.2 kcal/mol to 6.0 kcal/mol (Table S1).

Six structures present outstanding docking scores for 2RDopt than for 2RDT. Compounds **77**, and 119 present positive docking scores, suggesting an unfitting binding mode between the protein and the ligand. Analyzing these compounds, broad tails are the main reason for an increase in the docking scores (Fig. S3), which may suggest that for these compounds, the enzyme conformation, especially the random coil, must change its poition to allow a positive ligand-enzyme interaction. Docking scores obtained for 2RDopt and 2RDT were plotted against the pIC_50_ where no correlation between these values was found (Fig. S4). These results suggest that, at least for this case of study, that docking scores are not good predicting the pIC_50_ as reported in some works in the literature^62,63^. Still, docking is a powerful technique in discriminating compounds by their fitting in the active site and getting a first view of the pose and some interactions between the receptor and the ligand.

Ligand-enzyme interactions are crucial to inhibition, then potent compounds were chosen and their interaction with GO was evaluated. The binding mode and spot interactions on the three most potent compounds, determined by the ensemble model (82, 111, 116), and the three compounds with the lowest docking score (73, 81, 138) were analyzed in detail. Gl was also considered a control. As GO is an FMN-dependent enzyme, the interaction between GO and FMN was also analyzed along with the possible interactions between the ligands and FMN. Table 2 shows the experimental pIC_50_ values, docking scores, and the main interactions of the ligands with GO and FMN.

**Table 2:** Modelling and docking results obtained for the studied compounds.

Ligand	pIC_50_ exp	2RDopt docking score (kcal/mol)	#HB_FMN_	Interaction with GO	
				*HB*	*π*
73	6.38	− 12.2	2	Tyr26, Tyr132, Arg167, His260, Arg263	Trp110
81	5.66	− 11.9	2	Tyr26, Tyr132, Arg167, His260, Arg263	Trp110
82	7.23	− 9.1	2	Tyr26, Tyr132, Arg167, His260, Arg263	–
111	7.20	− 10.6	1	Tyr132, Arg167	Trp110
116	7.22	− 6.3	2	Tyr26, Tyr132, Met183, Arg263	Trp110
138	4.86	− 11.6	3	Tyr132, Arg167, Arg263	Trp110
Gl	–	− 4.3	2	Tyr26, Arg263	–

**Figure 7:**
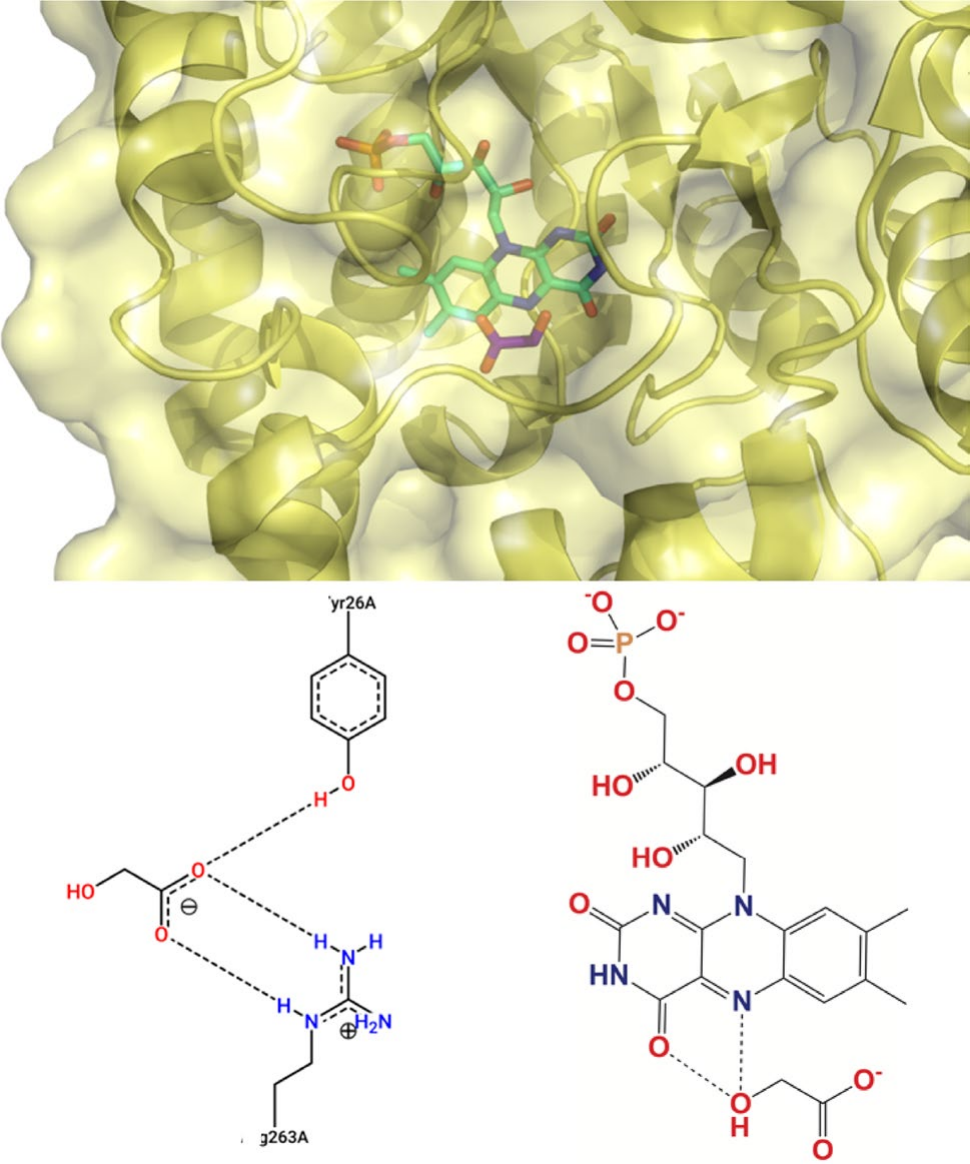
2D and 3D representation of the Interaction of Gl with FMN and GO.

As FMN is essential for Gl production, when 2RDU was analyzed (Fig. 7), two HBs between Gl and FMN were found. Furthermore, Gl forms HBs with Tyr26, and Arg263 in GO meaning these four interactions are responsible for catalyzing the glycolate-glyoxylate transformation. Looking at the studied molecules (Fig. S5, all of them present HBs with FMN where compound 111 produces 1 HB, compound 138 3 HBs, and the others 2 HBs. Interaction with Arg263 is present in all molecules except for compound **111**, while interaction with Tyr26 is only missing in compounds 111, and 138. Further analysis of compound 111 shows Tyr26 and Arg263 are just a little far to form HBs (4.2 Å and 3.9 Å respectively). As molecules are not static, this compound will likely form HBs with these two residues during GO inhibition emphasizing the importance of performing molecular dynamic studies after the docking analysis. Therefore, HBs with FMN, Tyr26, and Arg263 are also essential for GO inhibition agreeing with the literature^64,65^. As the studied compounds are bigger than Gl, other essential interactions can be found in structure size. All 6 compounds form HBs with Tyr132 while five compounds with Arg167; 3 compounds form HBs with His260, and one compound with Met183. Moreover, all compounds except 82 form a π interaction with Trp110.

To evaluate the stability of these bonds and to determine whether additional HBs could be formed during the interaction, MD simulations were performed.

### Molecular dynamics

**Figure 8:**
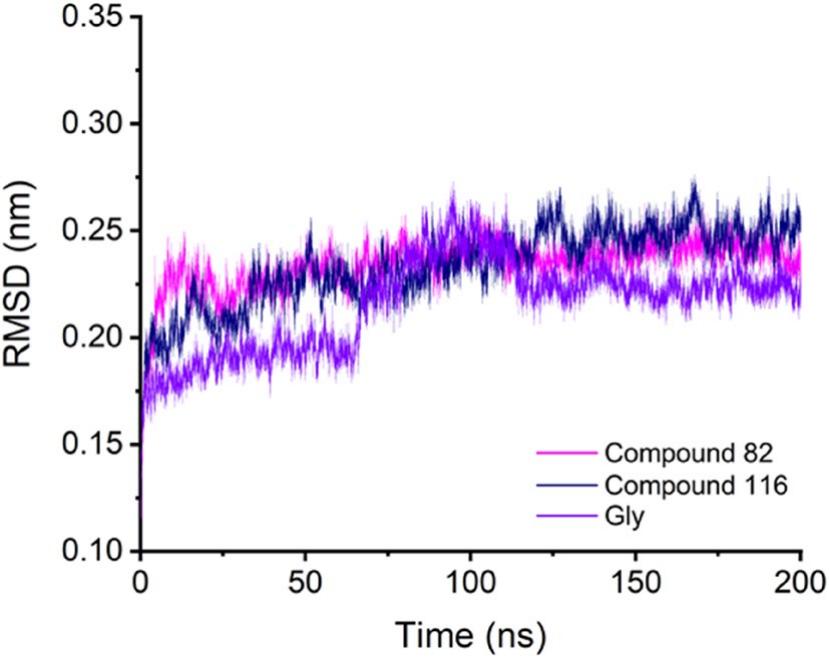
RMSD of GO during the 200 ns simulation.

To evaluate how compounds with different potencies interact with GO, ten compounds were chosen to guarantee the dataset representability in all the pIC_50_ spectrums, as follows. First, the compounds were ordered from highest to lowest pIC_50_ values; the two compounds having the highest pIC_50_ value (82 and 116) plus 7 compounds (4, 27, 39, 69, 100, 120, 124, and Gl) with medium and low potency for a broader analysis spectrum were randomly chosen. Then, a molecular dynamic simulation was performed for 200 ns using as input the ligand-FMN-2RDopt complex obtained from the docking calculations. Before analyzing all the trajectories obtained from the calculation and performing the analysis, a quality check was done to evaluate different parameters such as box size, temperature, volume, density, pressure, and potential energy during the 200 ns simulation without finding any odd behavior. The first step in the analysis was evaluating the stability of the different structures in the complex (ligand, FMN, and GO) via a root-mean-square deviation (RMSD) analysis. For clarity, only compounds 82, 116, and Gl are shown here in the main text, while the other compounds can be found in the Supporting Information. GO RMSD (Figs. 8 and S6) show a fast stabilization in all the studied systems (less than 5 ns) with values between 0.24 and 0.33 nm. These results suggest that the structure optimization produced 2RDopt was trustworthy.

**Figure 9:**
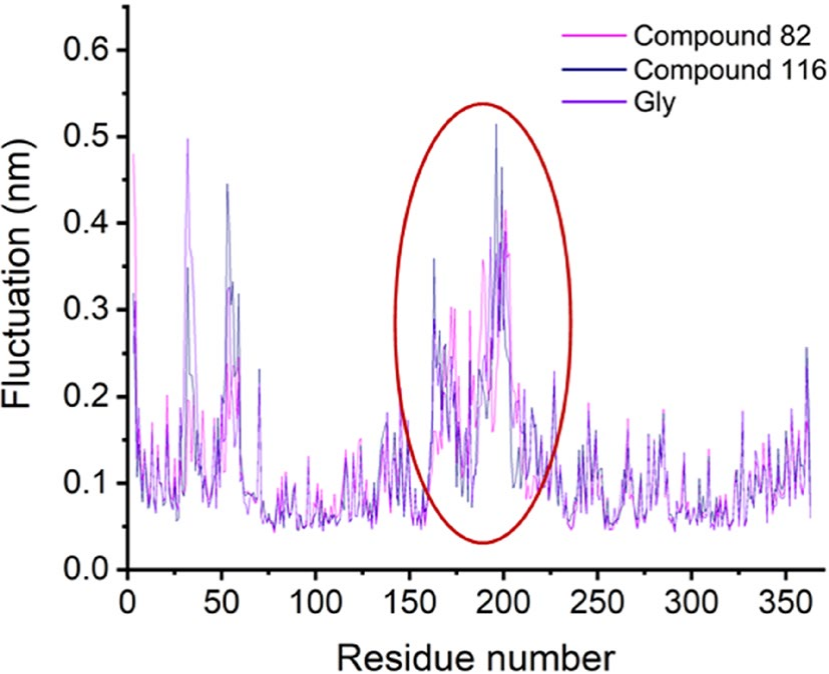
Root Mean Square Fluctuation (RMSF) of compounds 82, 116, and Gly. RC amino acids are presented inside the red circle.

The changes in RC were evaluated and compared between the different systems. Fluctuation analyses show amino acids Lys176 to Leu205 present the highest fluctuations during the simulation (Figs. 9 and S7). High fluctuations were also observed between Tyr50 and Ser63 where another external random coil is found. Interestingly, the system with the most significant fluctuation in RC (0.51 nm) has the biggest ligand (compound 116), suggesting the importance of the ligand size in RC fluctuation.

**Figure 10:**
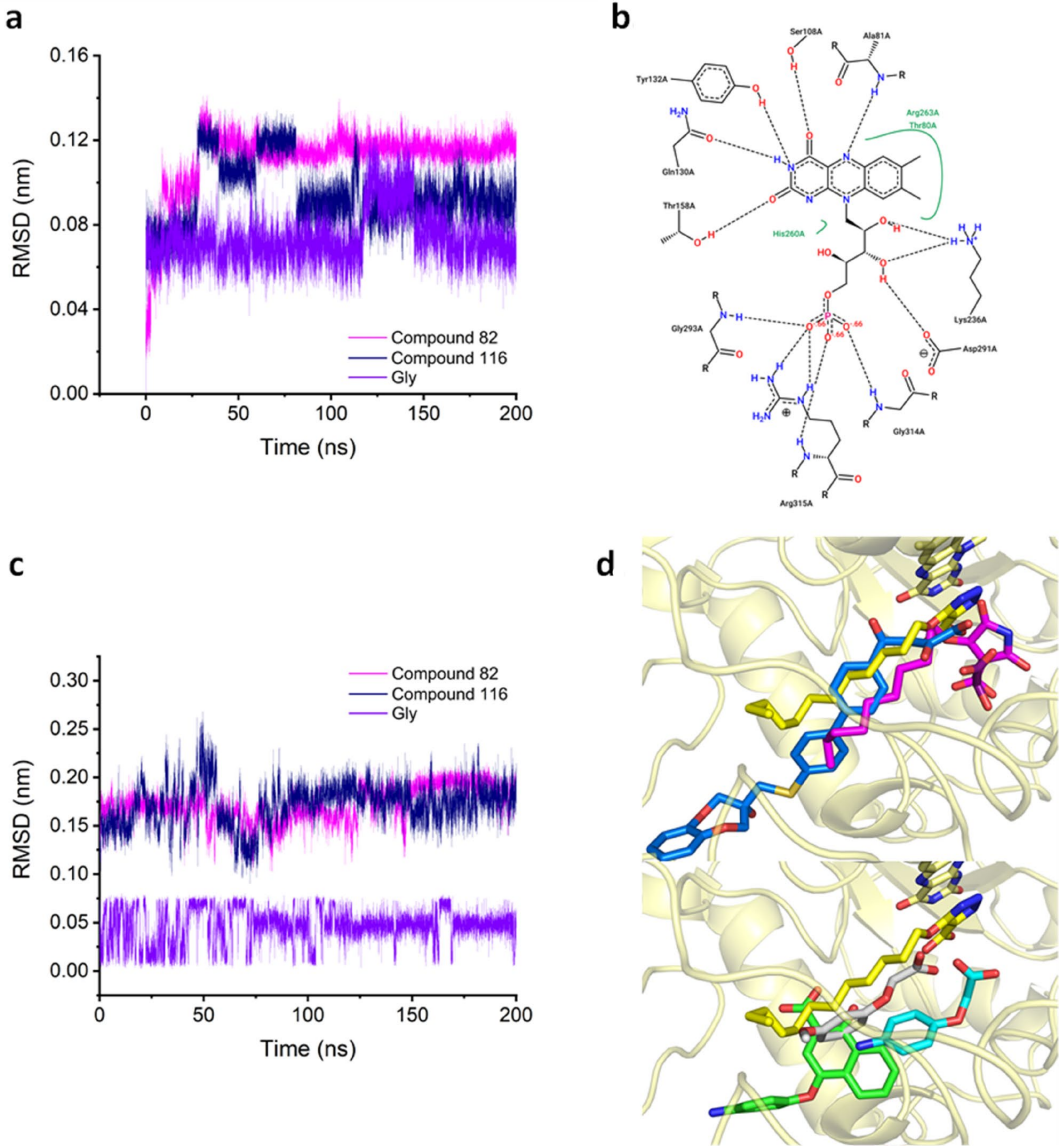
(**a**) RMSD of FMN during the 200 ns simulation. (**b**) 2D representation of the interaction between FMN and GO. (**c**) RMSD of the ligands during the 200 ns simulation. (**d**) Comparison of the structure after 200 ns simulation vs. experimental CDST (yellow).

The prosthetic group, FMN, is very stable inside GO presenting based on the RMSD that ranges between 0.02 nm and 0.14 nm (Figs. 10a and S8). Furthermore, when comparing the FMN position in 2RDU with the results of the MD simulation in all the systems, there is an overlap of the structures. Insights in FMN contact with GO show strong interactions that include 10 HBs (Fig. 10b). The RMSD of the ligands was also analyzed (Figs. 10c and S9a) where good stabilization throughout the simulations with values below 0.30 nm was achieved. Looking at the structure after the 200 ns, all the compounds stayed in the active site (Figs. 10d and S9b). Only compounds 27 (cyan), 39 (grey), and 124 (green) present a shift compared to the experimental CDST (2RDT ligand) and are located further from FMN than the other structure.

**Figure 11:**
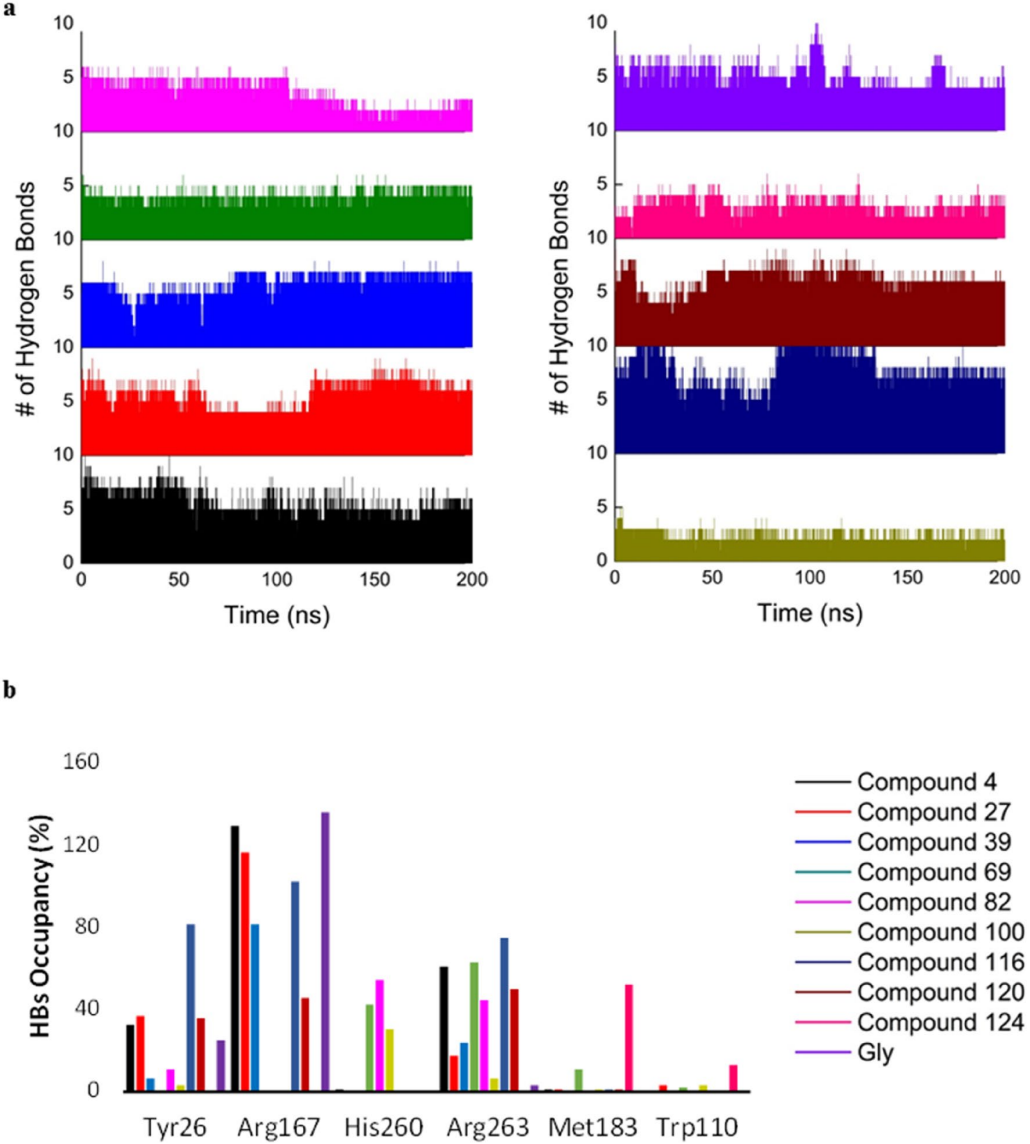
(**a**) HBs during the 200 ns simulation. (**b**) HBs occupancy of the studied compounds.

Hydrogen bonds (HB) and occupancies on each system were evaluated. As Fig. 11, all ten studied ligands form at least 1 HB during the 200 ns, which keeps the compounds in the active site during the simulation. Compound 116 forms the most HBs, with a maximum value of 13 and an average of 6, which agrees with its high potency (pIC_50_ = 7.22).

Occupancies demonstrate that the amino acids spotted to be important during the docking studies are crucial to forming strong interactions. The most essential amino acids are Tyr26, Arg167, and Arg263 being in concordance with the literature^64,65^. Arg167 presents the highest occupancies being present in 6 of the 10 compounds. Arg263 is present in all compounds except for 124, with percentages reaching 75% in compound 116. Analyzing the GO pocket, both arginine residues are next to each other, suggesting that, as the ligand interacts with the active side, HBs can be formed with either one. Compounds 4, 27, 39, 120, and 124 form high occupancy HBs with Gln264 (34%, 36%, 52%, 63%, and 71%, respectively), which may suggest this interaction important for inhibition. The results suggest that interactions with polar residues are favored during the inhibition, which agrees with the result found in the QSAR modeling, dominated mainly by the descriptors such as charges, electronegativity, etc.

Hydrophobic interactions also play a vital role in inhibition and help stabilize the ligand in the active site. In this sense, the results obtained from the docking and the molecular dynamics for the two compounds with the highest pIC50 (82 and 116) were analyzed and compared. For compound 82, while the docking result shows hydrophobic interactions of the ligand with Met82, Trp110, Met183, Phe193, Leu205, Tyr208, and Val209 (Fig. S5); most of these interactions are lost at the end of the molecular dynamics except Leu 205, Val209, and Trp110 (Fig. S10a). This result suggests that these three amino acids are key in inhibition but due to their ability to form hydrophobic interactions. Analyzing compound 116, these three amino acids present in the docking (Fig. S55), and only Val209 is lost in the molecular dynamics (Fig. S11b). Due to the big size of these molecules, Phe193 seems to be an important residue due to its appearance in both docking and molecular dynamics studies. Finally, let's analyze the compound with the lowest pIC_50_ (compound 27). It is observed that only one hydrophobic interaction (with Val209) is maintained at the end of the simulation (Fig. S10c), which is in may help explain its high pIC_50_ and binding energy values.

Finally, van der Waals, electrostatic solvent accessible surface area (SASA), and binding energies were calculated (Table 3). Binding energy show negative values for the compounds with higher pIC_50_ values and positive for lower pIC_50_ values. Binding energy results, calculated using the MMPBSA method, depend on several aspects, including charges in the cofactor, protein, and ligand and the dielectric constant^66,67^. Although these parameters can affect the accuracy of the results in terms of the values obtained, if the same parameters, protein, and cofactors are used, the results should be comparable to experimental values. Therefore, the experimental pIC_50_ value was plotted against the binding energy finding an excellent linear relationship with a determination coefficient (R^2^) of 0.81.

**Table 3:** van de Walls, electrostatic, SASA, and binding energy (BE) (in kcal/mol) of the studied compounds.

Compound	van der Waals energy	Electrostatic energy	SASA energy	Binding energy	pIC_50 Exp_
4	− 32.31	− 16.69	− 3.44	2.99	3.80
27	− 19.24	− 17.16	− 2.54	12.34	2.09
39	− 21.71	− 15.54	− 2.70	10.54	3.00
69	− 31.48	− 9.59	− 2.82	− 10.43	4.60
82	− 45.93	− 7.22	− 4.29	− 26.33	7.23
100	− 40.34	− 14.81	− 3.48	− 12.52	6.48
116	− 56.67	− 21.61	− 5.56	− 9.27	7.22
120	− 29.79	− 15.48	− 3.27	2.01	4.37
124	− 38.81	− 7.61	− 3.68	− 9.59	5.54

Furthermore, the binding energy was plotted against the predicted pIC_50_ obtained for the three models. A good correlation was also found with an R^2^ value of 0.78 for MLR1, 0.82 for RF1, and 0.84 for IBK1. These results agree with the literature where a lower binding energy value favors interactions between the ligand and GO, which directly increases inhibition potency (higher pIC_50_).

### Analysis of possible inhibitors extracted from the DrugBank database

**Figure 12:**
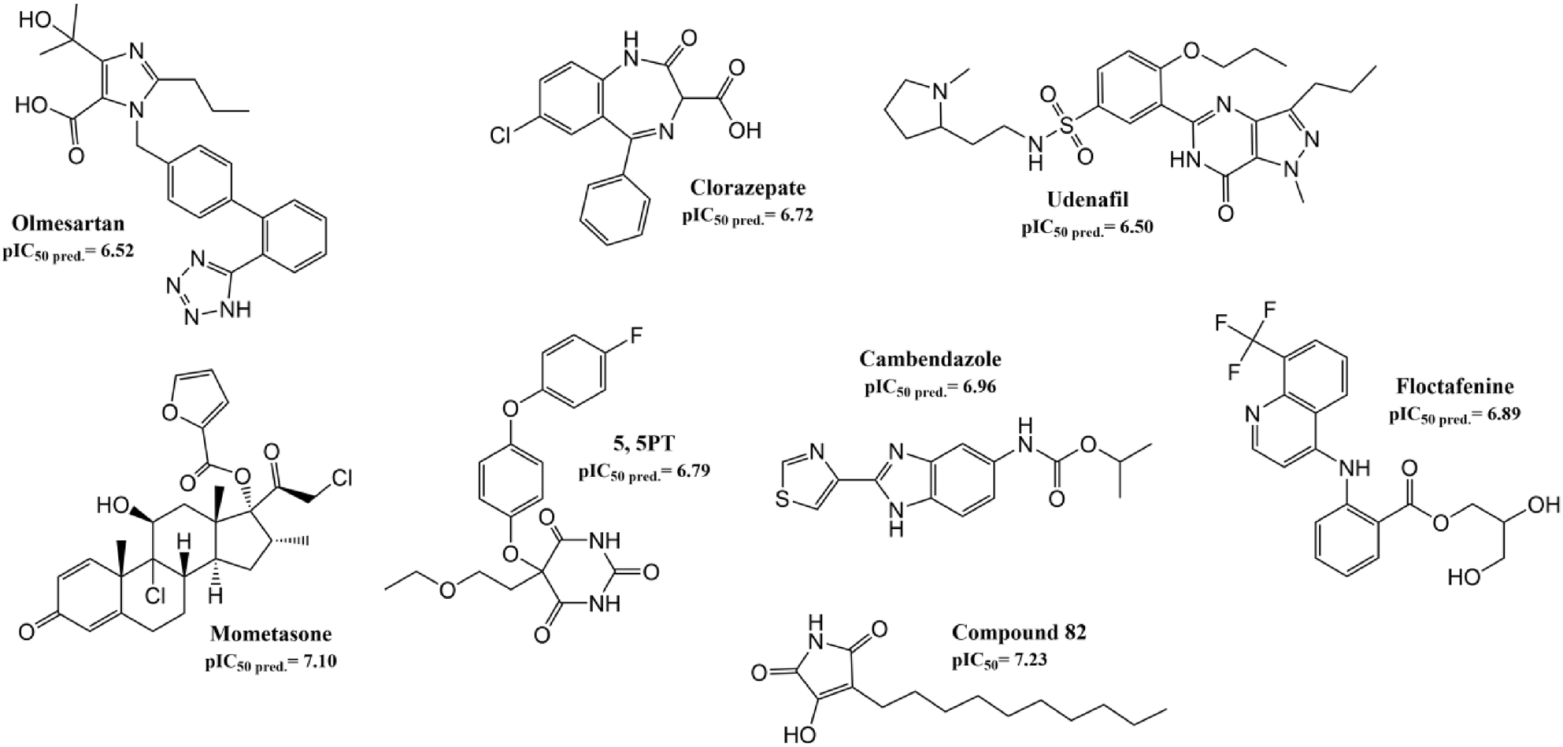
Chemical structure of the best molecules derived from the drug bank screening.

The seven candidates selected from the Databank were looked at more in detail and compared with compound 82 in their suggested ability to inhibit GO (Fig. 12). Interestingly, the seven molecules possess in the ends of their structure several heteroatoms (S, N, O) with the capacity of acting as hydrogen bond donors and acceptors, being able to interact with charged residues in the active site such as Arg167, His260, and Arg263; but also, with the hydroxyl group of Tyr26. Furthermore, in most cases, except for clorazepate, the structures present an extended structure that can mimic compound 82 hydrocarbon tail. This tail represents the main difference between compound 82 and the drugs from the screening. However, while compound 82 presents a hydrocarbon tail, the seven molecules present different moieties. These moieties include benzene rings that can form interesting π-π interactions with Trp110 (essential residue in the active site) and heteroatoms that can form hydrogen bonding at the end of the active site and in the tunnel conducting to it. On the other side, these characteristics may also present a drawback in inhibiting the compound from entering the active site or forming unfavorable interactions that can reduce the binding affinity. Therefore, molecular docking studies were performed to study the ligand-GO interaction more in detail and see the binding mode of the screened compounds.

Although all compounds present high predicted pIC_50_ values, positive docking scores (Table 4) in clorazepate, udenafil, and mometasone furoate infer that these molecules may not be able to fit in the active site. In this sense, the three-dimensional docking pose of the three molecules was analyzed in detail, noticing minor clashes in the outer part of the active site, where the RC is. This result suggests that for these molecules, either the RC will impede proper binding to the active site or the GO’s plasticity property and especially of the RC will rearrange its conformation to liberate those clashes allowing the three drugs to fit. On the contrary, negative docking scores and a binding mode without clashes were found for the other four molecules. Molecular dynamics were carried out to evaluate the interaction over time and study conformational changes in GO.

**Table 4:** Docking and molecular dynamic result of the Drug Bank Database screening.

Drug bank ID	Name	Docking score	Coulomb	LN	Binding energy
		kcal/mol			
DB00275	Olmesartan	− 3.2	− 17.09	− 54.63	− 33.94
DB00628	Clorazepate	1.9	− 10.74	− 38.94	− 19.32
DB06267	Udenafil	0.6	− 18.63	− 64.88	− 33.63
DB14512	Mometasone furoate	3.0	− 18.37	− 47.37	− 30.47
DB08388	5,5PT	− 7.1	− 15.53	− 44.31	− 27.52
DB11380	Cambendazole	− 9.3	− 12.46	− 38.34	− 27.29
DB08976	Floctafenine	− 7.6	− 12.75	− 43.67	− 23.24

**Figure 13:**
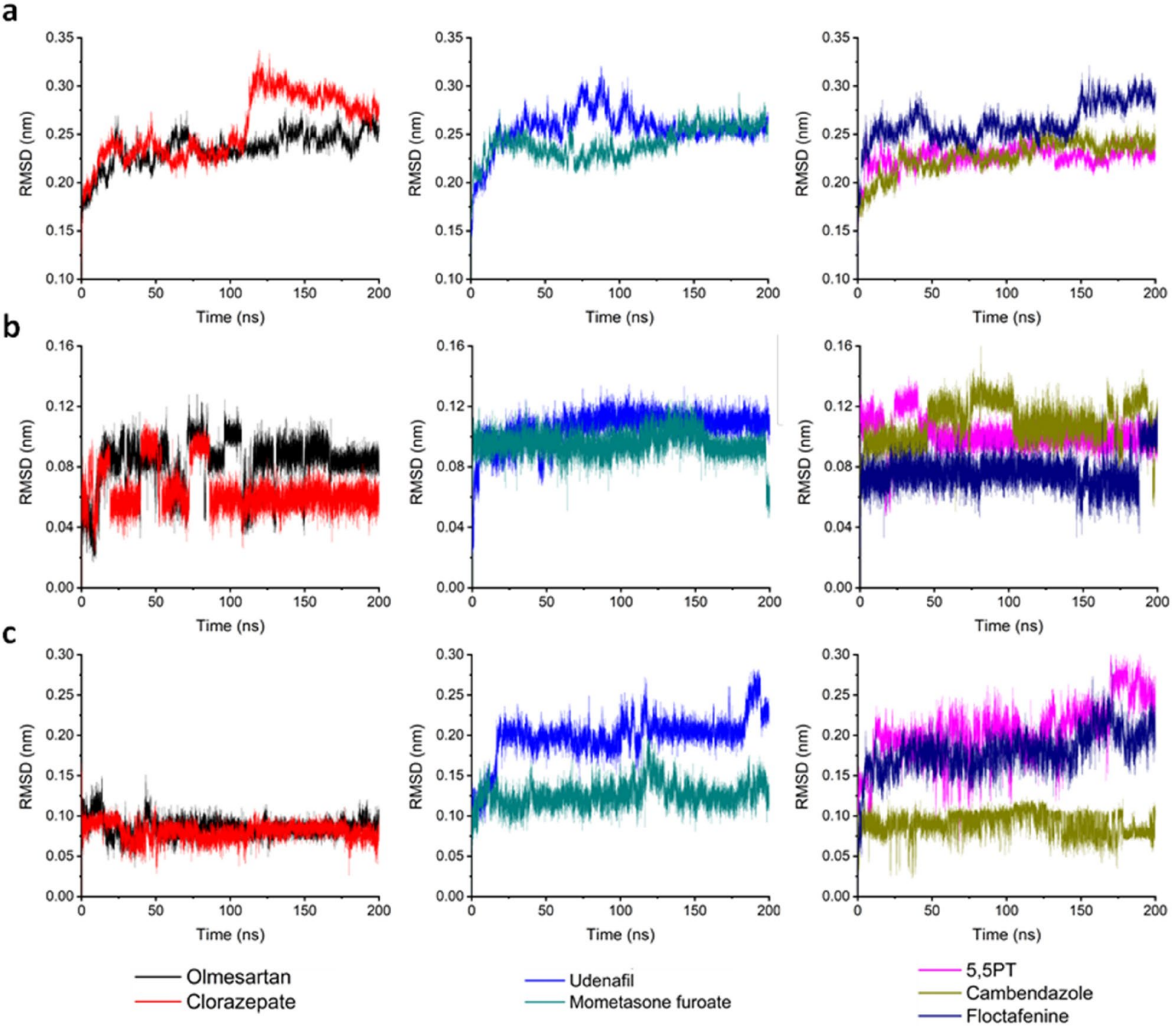
RMSD of GO (**a**), FCN (**b**), and the ligands (**c**) of the DB screening during the 200 ns simulation.

The analysis of the RMSD (Fig. 13) shows FCN, GO, and the ligands present the same behavior as the dataset systems with values for GO around 0.30 nm, for FCN between 0.02 and 0.17 nm, and the ligands below 0.30 nm.

The seven compounds form at least one HBs (Fig. S11) in most simulations. The maximum number of HBs formed in these compounds is six, although this happens only in olmesartan and floctafenine for a little time. On average, those seven compounds form 2 HBs, fewer HBs than the compounds in the dataset. This suggests that if these compounds manage to inhibit GO as their predicted pIC_50_ values indicate, forming only two HBs is enough to cause inhibition. Tessential HB occupancy of the seven compounds was analysed to determine which amino acids are key for possible GO inhibition (Fig. S11). Six out of the seven compounds present important occupancies with Tyr26 (from 1.1 to 55.4%), which is one of the essential amino acids for GO inhibition. The second most important amino acid is Trp110, with four compounds presenting high occupancies.

Interestingly, Trp110 has low occupancy with the data set molecules. This infers that some of these molecules may present a different binding mode involving other residues. Arg167 and Arg263, considered also as essential for GO inhibition in the data set, present an important occupancy only for floctafenine and mometasone furoate, respectively. Leu177 appears to be an important amino acid in this set of compounds having important occupancies in clorazepate (31.52%), 5,5PT (32.52%), and cambendazole (27.17%). In sildenafil, Leu191 and Leu205 are important, with occupancies of 36.96% and 48.92%, respectively. Finally, olmesartan presents a long-lasting hydrogen bond with Asp170 (33.7%).

An analysis of the whole trajectory of the molecular dynamic simulations shows that all the molecules studied managed to stay in the enzyme's active site, which is a first good indication of the possible inhibition property. Coulomb energies between − 10.74 and − 18.63 kcal/mol and Lennard-Jones potential values between − 38.3 and − 64.88 kcal/mol (Table 4) show a positive interaction between the drugs and GO, which is a second positive result towards the suggested inhibition of GO by these compounds. Finally, the binding energy estimated for the seven compounds reveals a possible favorable interaction between the ligands and GO. Binding energies (Table 4) range from − 19.32 kcal/mol in clorazepate and − 33.94 kcal/mol in olmesartan. The values are in accordance with the binding energy found for compound 82 (BE = − 26.33 kcal/mol), the most active compound of the training/test data set.

### ADMET properties and toxicity

**Figure 14:**
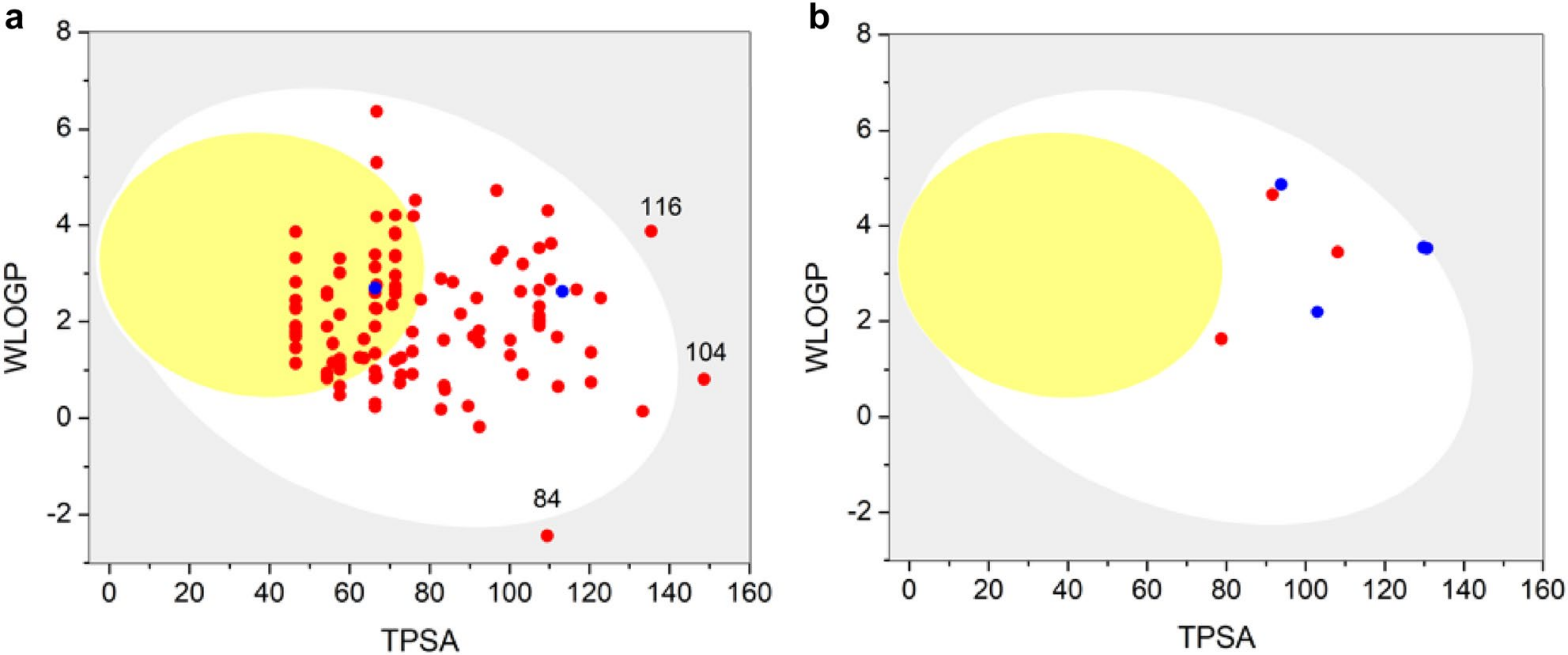
Boiled egg of (**a**) all the compounds in the database and (**b**) 7 best compounds from the screening showing probability of human intestinal absorption (HIA) and blood–brain barrier (BBB) permeation of the studied molecules.

ADMET properties were calculated for all the compounds in the database and the 7 best compounds through SwissADME (http://www.swissadme.ch (accessed on 27 July 2021)). The lipophilicity obtained from Wildman and Crippen method (WLOGP) and the topological polar surface area (TPSA) were used to construct the BOILED-Egg (Fig. 14). The plot WLOGP vs. TPSA represented as a boiled egg predicts brain penetration (yolk) and gastrointestinal absorption (white zone). Compounds 116, 84, and 104 in the grey region show poor intestinal absorption and brain penetration. P-glycoprotein substrates (Pg-p) is a restrictive barrier to maintaining homeostasis of the brain and is key among ATP-binding cassette transporters. P-glycoprotein substrates (red dots) and P-glycoprotein non-substrates (blue dots) are represented in plot^68^.

Lipophilicity and water solubility have facilitated drug formulation and handling^67^. Lipophilicity is expressed by the partition coefficient between n-octanol and water—called log Po/w. The solubility and lipophilicity of the compounds considered for molecular dynamics are shown (Table 5). For example, mometasone furoate can be considered too lipophilic based on its Log Po/w value (3.97). Three models evaluated water solubility: 1) ESOL^69^; 2) from Ali et al.^70^; 3) developed by SILICOS-IT^71^. The compounds—except for 69—showed either very soluble (Vs), moderately soluble (Ms), or soluble (S) in at least two methods.

**Table 5:** Properties calculated from ADME, solubility (S = Soluble, Ms = Moderately soluble, Ps = Poorly soluble), and log Po/w consensus.

Name	ESOL class	Ali class	Silicos-IT class	Log Po/w
Olmesartan	Ms	Ms	Ps	3.08
Clorazepic acid	Ms	Ms	Ms	2.32
Idenafil	Ms	Ms	Ps	3.03
Mometasone furoate	Ms	Ms	Ps	3.97
Ethoxyetyl	S	Ms	Ps	2.4
Cambendazole	S	Ms	Ms	2.65
Floctafenine	Ms	Ms	Ps	3.36
Compound 4	S	S	S	2.03
Compound 27	Vs	S	S	0.72
Compound 39	Vs	S	S	1.2
Compound 69	Vs	Vs	S	0.19
Compound 82	S	Ms	Ms	2.97
Compound 100	S	S	S	0.76
Compound 116	Ms	Ps	Ps	3.33
Compound 120	S	S	S	0.96
Compound 124	Ms	Ms	S	2.45

## Materials and methods

### Dataset and descriptors

The molecular structure and the GO inhibitory activity [pIC_50_ = − log(IC_50_,M)] of 144 GO inhibitors were obtained from the literature^10–13^ and used as the dataset for this study. The 3D molecular structures of these molecules were drawn using GaussView (Version 5.0) followed by their optimization at the molecular mechanic level Universal Force Field (UFF)^72^ using Gaussian 16 suite^73^. The self-consistent field (SCF) convergence criterion was set as default, and a frequency calculation for all structures was performed to confirm that they are minimum stationary points^74^. The simplified molecular-input line-entry system (SMILES) with their corresponding pIC_50_ of the molecules is available in the supporting information (Table S1). Topographic descriptors were used for the modelling and calculated through QuBiLs-MAS (2D-descriptors)^75^ and QuBiLs-MIDAS (3D-descriptors)^29^. These descriptors have demonstrated a good capacity for building models to predict pIC_50_ values^32–36^.

### QSAR modeling process and descriptor selection

The descriptor selection approach increases the accuracy and reduces overfitting due to irrelevancy and redundancy. The Wrapper method selects appropriate descriptors for a given subset and can be used with various machine learning algorithms^76^. The Wrapper method is a black box that explores the space to rank the features based on their predictive power by adding or removing them from the given subset ^77,78^. Individual models were obtained with the wrapper method using Weka 3.8^79^ with three widely described regression techniques^20^: multiple linear regression (MLR), instance-based learning with parameter k (IBK), and random forest (RF). The dataset was split into training (75%) and test (25%) sets with K-means clustering analysis, which divides the data into “K” non-hierarchical number of clusters^80^.

### Applicability domain

The applicability domain is the theoretical space based on the training set of a model, where QSAR predictions are reliable. The applicability domain is defined by the descriptors of the model^81,82^. Defining the applicability domain allows for identifying outliers in the test set, which are the compounds outside the domain whose prediction cannot be considered reliable. We defined the applicability domain with the descriptors of the three most robust models. We used a consensus of four methods—Range, Euclidean distance, City-block distance, and probability density—implemented in AMBIT software^83^. All outliers, if any, must be taken out of the study, and the statistical parameters of all the models recalculated. A molecule will be removed if it is defined as an outlier in two or more methods.

### Model performance

The quality of the model was evaluated under goodness-of-fit and goodness-of-prediction parameters. Goodness-of-fit parameters measure the degree of conformity of the sample data to the model^84^. Therefore, goodness-of-fit evaluates the ability of the model to explain the variance observed in the training set. The statistics implemented to evaluate goodness-of-fit were the mean absolute error (MAE) and the coefficient of determination (R^2^)^85^. The goodness-of-prediction parameters measure the model's predictive power through a tenfold cross-validation coefficient (Q^2^_CV_) and through the prediction of external compounds not considered to train the model denoted as the external validation coefficient (Q^2^_EXT_)^86^.

### Ensemble model

An ensemble model was obtained to improve the prediction level of individual models^87^. Ensemble models reduce errors due to the sample size and over-fitting during the training phase related to weak descriptors^88^. In this study, the ensemble model was constructed by averaging the predicted pIC_50_ values obtained from the best individual models.

### DrugBank screening

The DrugBank database (V.5.1.7; http://www.DrugBank.ca/) was used for the in silico screening. These drugs were downloaded and optimized to calculate the 3D-molecular descriptors. All the drugs within the applicability domain were evaluated as GO inhibitors by predicting their pIC_50_. The most potent candidates were considered for molecular docking and molecular dynamics studies.

### Molecular docking

Molecular docking is one of the most used techniques in drug design for its capacity to predict the binding mode of a compound in the active site of an enzyme^89^. All 144 compounds of the dataset and Gl (positive control)—now called ligands—were docked against the enzyme. The enzyme 2RDopt was built using the X-ray diffraction crystal structure of human glycolate oxidase in a complex with glyoxylate (PDB: 2RDU) and with 4-carboxy-5-dodecylsulfanyl-1,2,3-triazole (CDST) (PDB: 2RDT)^65^. 2RDopt was created to preserve the enzyme's three-dimensional conformation when interacting with an inhibitor (2RDT) and the random coil motif elucidated in 2RDU. To prepare 2RDopt for the calculations, 2RDT and 2RDU were first aligned. Gl was deleted from 2RDU and replaced by CDST. In the 2RDU-CDST complex, several clashes between the tail of CDST and the random coil were noticed. Therefore, an optimization of the new complex was done. For this, water molecules were removed from the complex, along with other residues not belonging to the system. The optimization was performed using the YASARA Energy Minimization Server^90^. After that, the CDST ligand was removed from 2RDopt using PyMOL (v1.8)^91^.

AutoDock Vina was used for all the docking calculations using a 1 Å spacing, default exhaustiveness, and full ligand flexibility for this study. AutodockTools^92^ was used to add polar hydrogens and obtain the structure in .PDBQT format. Flavin mononucleotide (FMN) was kept during the process as it is a prosthetic group in GO, which is crucial for glycolate transformation. The coordinates used for the calculations were determined based on the active site elucidated by experimental crystallographic structures^65^. The grid box was reduced to the active site (x = 36.711, y = 7.152, and z = 11.558) with a size of 18 Å for x, and 14 Å for y and z, respectively. As criteria for this analysis, the docking results were assessed considering the docking score, the hydrogen bonds formed with FMN and the different hydrophobic and polar interactions occurring between the ligand and the protein.

### Molecular dynamics

Molecular dynamics (MD) simulation is a powerful tool to evaluate ligand-enzyme complex stability in time. Results provide insights into the interactions occurring in the complex, including hydrogen bonds (HBs) formation and interaction energies. For the MD simulations, 10 compounds were selected based on the pIC_50_. For the selection, the dataset was ordered from highest to lowest pIC_50_ values; the two compounds having the highest pIC_50_ value (compounds 82 and 116) plus 7 other compounds taken randomly from the middle and lower part of the list i.e. compounds 4, 27, 39, 69, 100, 120, 124, and Gl as control compound. The complex resulting from the docking calculations was chosen as starting structure for the simulation. The topology of GO was built using AMBER99SB-ILDN^93^ implemented in Gromacs 2019, while the topology of the ligands used ACPYPE server and Generalised Amber Force Field (GAFF). A cube shape system solvation with the three-point water model (TIP3) was used. Then, the system was neutralised using sodium or chlorine atoms as needed. The complex was relaxed and then equilibrated for 100 ps at 300 K using a constant number of particles, volume, and temperature (NVT) in the first part and a constant number of particles, pressure, and temperature (NPT) in the second one^94^. All simulations were run for 200 ns, 1 Barr, and 300 K^95^ using Gromacs 2019 software^96^. From the simulation, the box size, volume, temperature, pressure, density, and potential energy ensure no errors occurred during the calculation. Furthermore, parameters such as the root-mean-square deviation, fluctuation, number of hydrogen bonds, hydrogen bonds occupancies, Lennard-Jones potential, Coulomb energy, and different interactions between the protein and the ligands were considered for the analysis.

All the studied models' free energy of binding was calculated using the Molecular Mechanics Poisson-Boltzmann Surface Area (MM-PBSA) method. Binding free energy is calculated from the sum of the vacuum molecular mechanics potential energies obtained from bonded and non-bonded interactions (EMM), plus the polar solvation energy calculated by solving the Poisson-Boltzmann equation (Gpolar), plus the non-polar solvation energy obtained using the Solvent Accessible Surface Area (SASA) model (Gnonpolar) as stated in Eq. (1).

1 $${\text{G}}_{{\text{X}}} = {\text{ E}}_{{{\text{MM}}}} + {\text{ G}}_{{{\text{polar}}}} + {\text{ G}}_{{{\text{nonpolar}}}}$$

These energies were obtained for the protein, the ligand, and the complex (X) using g_mmpbsa tool taking snapshots every 5 ns in the trajectory between 10 and 200 ns.

**Figure 15:**
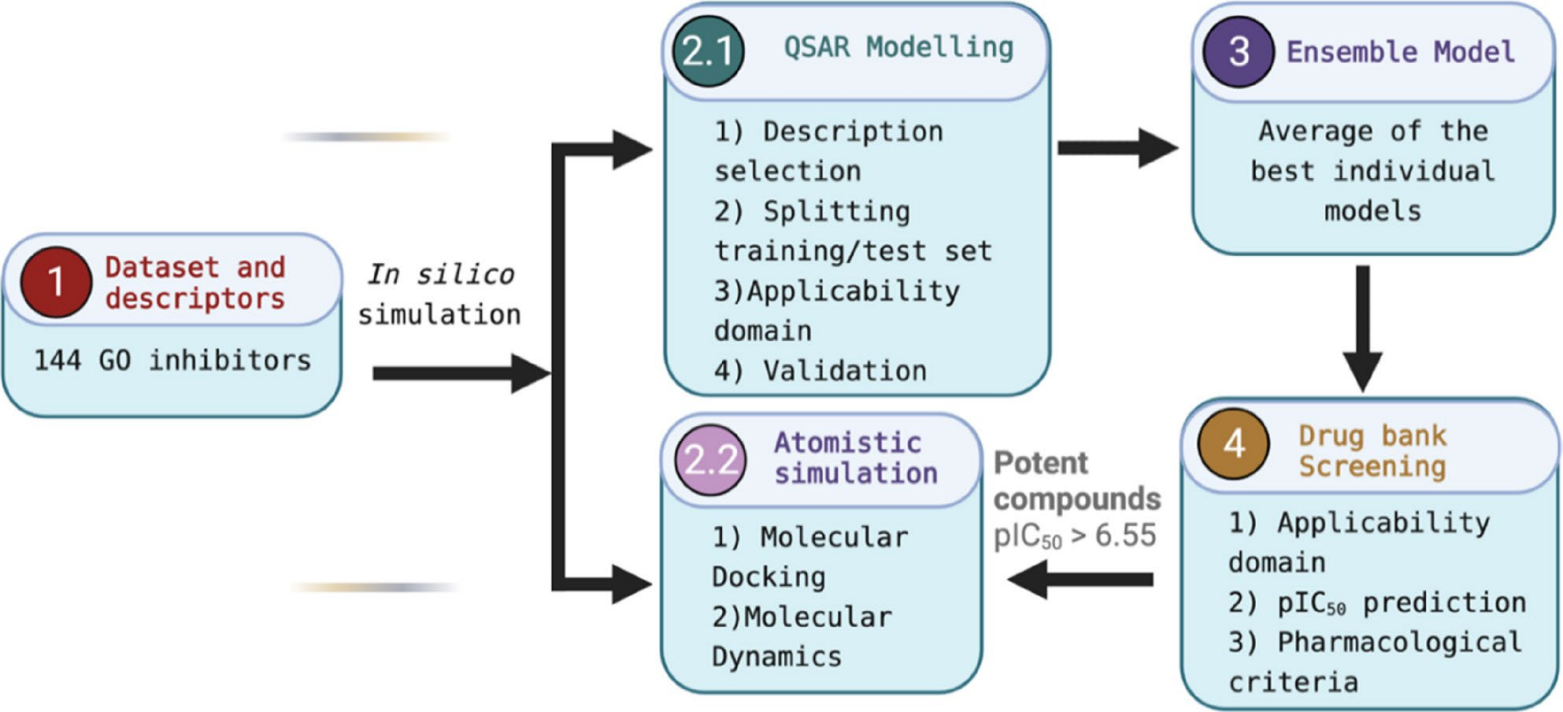
Flow diagram for the applied methodology in this work.

A schematic representation of the applied methodology is presented in Fig. 15, showing the workflow step by step.

## Conclusions

Three models (MLR1, RF1, and IBK1) with good predictability performance, robust enough, and with $\le$ 8 descriptors were obtained based on the determination coefficients (> 0.87) for the training the tenfold cross-validation and external validation. An unsupervised ensemble, obtained by averaging the predictions of the three models, enhances the predictability represented by a high Q^2^_EXT_ of 0.933. The descriptors present in the models are weighed by physicochemical properties, which characterize the polar nature of the functional groups present in the molecular core (carboxylic acid, alcohols, amine, etc.). Docking analysis agrees with these results, where negative docking scores were found, and predominant interactions with polar residues were observed. Molecular dynamics simulations were performed for 200 ns to elucidate ligand-enzyme interactions mainly based on hydrogen bond formation and their occupancies. QSAR, molecular docking, and molecular dynamic approaches were employed to find alternative drugs from the DrugBank database. A screening of the total database with the QSAR model, a subsequent evaluation of the AD, molecular docking and molecular dynamics analysis of the best candidates; led to the proposal of 7 well know compounds [Olmesartan, benzodiazepine clorazepate, udenafil, mometasone furoate, 5-(2-Ethoxyethyl)-5-[4-(4-fluorophenoxy)phenoxy]pyrimidine-2,4,6(1H,3H,5H)-trione (5,5PT), cambendazole, and floctafenine] as possible GO inhibitors. These molecules are suggested to be tested as GO inhibitors or used as lead compounds for the design of PHT1 treatments.

## Supplementary Information

Supplementary Information.

## Data Availability

All data generated or analyzed during this study are included this article and its supplementary information file.
