# Supplementary material for: Searching glycolate oxidase inhibitors based on QSAR, molecular docking, and molecular dynamic simulation approaches
Source: Sci Rep. 2022 Nov 19;12:19969. doi: 10.1038/s41598-022-24196-4 (PMC9675741; doi:10.1038/s41598-022-24196-4)
Supplement: Supplementary file 1 — Supplementary Information. [file 41598_2022_24196_MOESM1_ESM.pdf]

Searching Glycolate Oxidase Inhibitors Based on QSAR, Molecular Docking, and Molecular Dynamic Simulation Approaches.

Nicolás Cabrera [1], Sebastián A. Cuesta [2,3], José R. Mora [3,*], José Luis Paz [4], Edgar A. Márquez [5,*], Patricio J. Espinoza-Montero [6], Yovani Marrero-Ponce [7,8], Noel Pérez [9], Ernesto Contreras-Torres[10]

[1] Department of Biomedical Engineering, Texas A&M University, College Station, Texas 77843, USA.
[2] Department of Chemistry, Manchester Institute of Biotechnology, The University of Manchester, 131 Princess Street, Manchester, M1 7DN, UK.
[3] Instituto de Simulación Computacional (ISC-USFQ), Departamento de Ingeniería Química, Universidad San Francisco de Quito, Diego de Robles y Vía Interoceánica, Quito 170901, Ecuador
[4] Departamento Académico de Química Inorgánica, Facultad de Química e Ingeniería Química, Universidad Nacional Mayor de San Marcos, Lima, Perú.
[5] Grupo de Investigaciones en Química y Biología, Departamento de Química y Biología, Facultad de Ciencias Básicas, Universidad del Norte, Carrera 51B, Km 5, vía Puerto Colombia, Barranquilla 081007, Colombia.
[6] Escuela de Ciencias Químicas, Pontificia Universidad Católica del Ecuador, Quito 17012184, Ecuador.
[7] Universidad San Francisco de Quito (USFQ), Grupo de Medicina Molecular y Traslacional (MeM&T), Colegio de Ciencias de la Salud (COCSA), Escuela de Medicina, Edificio de Especialidades Médicas, Diego de Robles y vía Interoceánica, Pichincha, Quito 170157, Ecuador.
[8] Departamento de Ciencias de la Computacion, Centro de Investigacion Cientifica y de Educacion Superior de Ensenada (CICESE), Ensenada, Baja California, Mexico.
[9] Universidad San Francisco de Quito (USFQ), Colegio de Ciencias e Ingenierías "El Politécnico", Quito 170901, Ecuador.
[10] BCAM – Basque Center for Applied Mathematics, Mazarredo 14, E48009, Bilbao, Basque Country – Spain

Supplementary information

Index	Page
**Tables**	
**Table S1.** SMILES with their corresponding pIC_{50}, training and test label and docking results for possible glycolate oxidase inhibitors of the molecules	1
**Table S2.** R^2_{ADJ}, Q^2_{EXT}, Q^2_{CV}, number of features and MAE obtained for the best nine models	6
**Table S3.** Name and abbreviation of the features of the 3 most robust individual models	6
**Table S4.** Collinearity for the MLR1's features	7
**Table S5.** Collinearity for the RF1's features	7
**Table S6.** Collinearity for the IBK1's features	7
**Figures**	
**Figure S1.** Number of descriptors, 10-fold cross validation coefficients, and MAE values obtained considering the whole dataset for the 9 individual models obtained in the first screening	8
**Figure S2.** a. 3D structure alignment of 2RDU (yellow), 2RDT (green), 2W0U (pink), and 2RDopt (grey). b. 2RDU and 2RDT showing the missing RC (fuchsia). c. 2RDU-CDST complex before (left) and after (right) the optimization. d. RC comparison of 2RDU before (fuchsia) and after (purple) the optimization	8
**Figure S3.** Docking results for compound 77 (a) and 119 (b)	9
**Figure S4.** Experimental pIC_{50} versus 2RDT (a) and 2RDopt (b) docking scores	9
**Figure S5.** 2D representation of the interactions of the studied compounds with FMN and GO	11
**Figure S6.** RMSD of GO during the 200 ns simulation	12
**Figure S7.** Root Mean Square Fluctuation (RMSF) of studied compounds	12
**Figure S8.** RMSD of FMN during the 200 ns simulation	13
**Figure S9. a**. RMSD of the ligands during the 200 ns simulation. **b**. Comparison of compound 4 (light green), 69 (dark green), 100 (wheat), and 120 (pink) after 200 ns simulation vs experimental CDST (yellow)	14
**Figure S10.** 2D representation of the interactions of compound 82 (a), 116 (b), and 27 (c) GO.	14
**Figure S11.** Number of hydrogen bonds between GO and Olmesartan (a), clorazepate (b), udenafil (c), mometasone furoate (d), 5,5PT (e), cambendazole (f), and floctafenine (g) during the 200 ns simulation and their occupancies.	16

Table S1 . SMILES with their corresponding experimental pIC_{50}, training and test label, and docking results for possible glycolate oxidase inhibitors of the molecules

Molecules ID	SMILES	pIC_{50}	Docking score (kcal/mol)		Label	Ref.
			2RDT	*2RDopt*		
1	O[C@@H](c1ccccc1)C(=O)O	2.4	-5.9	-7	Training	1
2	O[C@@H](c1ccc(cc1)I)C(=O)O	3.79	-6.1	-7.3	Training	1
3	O[C@@H](c1ccc(cc1)Cl)C(=O)O	3.23	-6.1	-7.5	Test	1
4	OC(=O)[C@H](c1ccc(cc1)Oc1ccccc1)O	3.8	-7.1	-9.8	Training	1
5	OC(=O)[C@H](c1ccc(cc1)c1ccccc1)O	4.4	-8.1	-10.3	Test	1
6	O[C@@H](c1ccc(cc1)F)C(=O)O	2.4	-6	-7.3	Test	1
7	O[C@@H](c1ccc(cc1)n1cccc1)C(=O)O	4.16	-6.8	-8.8	Training	1
8	O[C@@H](c1ccc(cc1)Br)C(=O)O	3.39	-6.1	-7.5	Training	1
9	OC(=O)[C@H](c1cccc(c1)N(=O)=O)O	2.75	-6.1	-7.6	Test	1
10	OC(=O)[C@H](c1ccccc1Cl)O	2.77	-4.7	-6.9	Training	1
11	CCSC[C@@H](C(=O)O)O	2.72	-4.5	-5.4	Test	1
12	O[C@H](C(=O)O)Cc1ccccc1	3.79	-6.2	-7.8	Training	1
13	O[C@H](C(=O)O)/C=C\c1ccccc1	3.33	-6.6	-7.8	Training	1
14	O[C@H](C(=O)O)C1CCCCC1	2.4	-5.4	-7	Training	1
15	O[C@H](C(=O)O)CC(C)C	2.6	-5.1	-5.9	Test	1
16	O[C@H](C(=O)O)CSc1ccc(cc1)c1ccccc1	5	-7.5	-10.7	Training	1
17	CSCC[C@@H](C(=O)O)O	2.3	-4.4	-5.4	Training	1
18	C[C@H]1CCC[C@@H](C[C@@H](C[C@@H](C1)C)C)[C@@H](C(=O)O)O	3.4	-5	-3.7	Test	1
19	OC(=O)COc1ccccc1	2.74	-5.5	-7.1	Training	1
20	OC(=O)COc1ccc(cc1)N(=O)=O	2.96	-5.7	-7.6	Training	1
21	OC(=O)COc1ccc(cc1)C(C)(C)C	3.64	-6.6	-8.9	Test	1
22	OC(=O)COc1ccc(cc1)O	2.64	-5.9	-7.2	Training	1
23	OC(=O)COc1ccc(cc1)Cl	3.8	-5.6	-7.6	Training	1
24	COc1ccc(cc1)OCC(=O)O	2.64	-5.7	-7.5	Training	1
25	OC(=O)COc1ccc(cc1)c1ccccc1	3.8	-7.6	-10.4	Test	1
26	OC(=O)COc1ccc(cc1)/C=C/C(=O)C	3.85	-6.6	-8.9	Test	1
27	OC(=O)COc1ccc(cc1)N	2.09	-5.9	-7.2	Training	1

28	OC(=O)COc1ccc(cc1)/C=C/N(=O)=O	3.14	-6.2	-8.6	Training	1
29	OC(=O)COc1ccc(cc1)C(CC(C)(C)C)(C)C	3.27	-7.1	-8.7	Training	1
30	OC(=O)COc1ccccc1C(=O)N	2.57	-4.9	-7	Training	1
31	OC(=O)COc1ccccc1C	3.55	-5.3	-7.3	Training	1
32	OC(=O)COc1ccccc1Cl	3.27	-5.2	-7.1	Test	1
33	OC(=O)COc1ccccc1N(=O)=O	2.49	-4.9	-7	Training	1
34	COc1ccccc1OCC(=O)O	2.8	-5.2	-6.7	Test	1
35	OC(=O)COc1ccccc1Br	2.31	-5	-6.7	Test	1
36	OC(=O)COc1ccccc1O	2.62	-5.5	-7	Training	1
37	C=CCc1ccccc1OCC(=O)O	3.8	-5.5	-7.2	Training	1
38	C/C=C/Cc1ccccc1OCC(=O)O	3.82	-5.9	-7.7	Training	1
39	COc1cccc(c1)OCC(=O)O	3	-5.4	-7.5	Training	1
40	OC(=O)COc1cccc(c1)Cl	3.43	-5.5	-7.6	Training	1
41	OC(=O)COc1cccc(c1)C	3.57	-5.9	-7.6	Training	1
42	OC(=O)COc1cccc(c1)I	3.35	-5.6	-7.5	Training	1
43	OC(=O)COc1cccc(c1)NC(=O)C	2.28	-5.8	-8	Test	1
44	OC(=O)COc1cccc(c1)N(=O)=O	3.09	-5.8	-7.8	Training	1
45	OC(=O)COc1cccc(c1)C(F)(F)F	3.46	-6.4	-8.7	Training	1
46	CCOc1cccc(c1)OCC(=O)O	3.09	-5.6	-7.9	Training	1
47	OC(=O)COC/C=C/c1ccccc1	2.57	-7	-8.8	Training	1
48	OC(=O)COc1ccc2c(c1)cccc2	3.09	-7.1	-9.7	Training	1
49	OC(=O)COCc1ccccc1	3.1	-6	-7.6	Training	1
50	OC(=O)C(=O)c1ccc(cc1)c1ccccc1	3.21	-7.8	-10.5	Training	1
51	O=C(c1ccccc1)C(=O)O	2.64	-5.8	-7.4	Training	1
52	OC(=O)C(=O)Cc1cc(OCc2ccccc2)ccc1N(=O)O	4.19	-7.6	-8.4	Training	1
53	O=C(C(=O)O)Cc1c(C)cccc1N(=O)=O	4.02	-4.6	-6.3	Test	1
54	COc1cc(ccc1OC)CC(=O)C(=O)O	3.85	-5.8	-7.7	Training	1
55	O=C(C(=O)O)Cc1ccccc1	4.12	-6.2	-8.1	Training	1
56	OC(=O)C(=O)Cc1ccccc1N(=O)=O	4.37	-5	-7.1	Training	1
57	COc1ccc(cc1O)CC(=O)C(=O)O	3.64	-6.1	-8.4	Test	1
58	O=C(C(=O)O)/C=C/c1ccc(cc1)Cl	3.57	-7.2	-9.3	Training	1
59	COc1cccc(c1)/C=C/C(=O)C(=O)O	3.4	-7.1	-9	Training	1
60	OC(=O)C(=O)/C=C/c1ccc(c(c1)Cl)Cl	4.1	-7.3	-9.5	Training	1

61	Clc1ccc(c(c1)Cl)/C=C/C(=O)C(=O)O	4.1	-6.8	-8.7	Test	1
62	OC(=O)C(=O)/C=C/c1ccccc1Cl	3.8	-6.5	-8.5	Training	1
63	COc1cc(/C=C/C(=O)C(=O)O)ccc1OC	2.92	-7.1	-8.7	Training	1
64	CCCCC(=O)C(=O)O	4	-5	**-6**	Training	1
65	O=C1NC(=O)C(=C1c1ccccc1)O	4.87	-5.8	**-9.3**	Training	2
66	Brc1ccc(cc1)C1=C(O)C(=O)NC1=O	5.6	-6.5	-9.9	Training	2
67	Ic1ccc(cc1)C1=C(O)C(=O)NC1=O	5.7	-6.3	-9.8	Test	2
68	O=C1NC(=O)C(=C1c1ccc(cc1)N(=O)=O)O	5.10	-6.2	-9.8	Training	2
69	Nc1ccc(cc1)C1=C(O)C(=O)NC1=O	4.60	-6.1	-9.2	Training	2
70	O=C1NC(=O)C(=C1Cc1ccccc1)O	5.03	-6.4	-9.6	Test	2
71	O=C1NC(=O)C(=C1O)c1ccc(cc1)C1CCCCC1	5.98	-7.3	-11.2	Training	2
72	O=C1NC(=O)C(=C1O)c1ccc(cc1)[C@]12C[C@H]3C[C@@H](C2)C[C@@H](C1)C3	5.74	-8.3	-9.4	Training	2
73	O=C1NC(=O)C(=C1O)c1ccc(cc1)c1ccccc1	6.38	-8.3	-12.2	Training	2
74	Brc1ccc(cc1)c1ccc(cc1)C1=C(O)C(=O)NC1=O	7.06	-8.1	-10.7	Training	2
75	O=C1NC(=O)C(=C1c1ccc(cc1)Cc1ccc(c(c1)Cl)Cl)O	6.66	-8.2	-11.4	Test	2
76	O=C1NC(=O)C(=C1c1ccc(cc1)[C@@H]1CCCc2c1cccc2)O	5.89	-7.6	-9.7	Training	2
77	O=C1NC(=O)C(=C1c1ccc(cc1)n1c(cc2c1cccc2)c1ccccc1)O	6.30	-7.3	6	Training	2
78	O=C1NC(=O)C(=C1C(=O)c1ccc(cc1)c1ccccc1)O	4.76	-7.9	-11.5	Training	2
79	O=C1NC(=O)C(=C1Oc1ccc(cc1)c1ccccc1)O	6.57	-7.6	-10.1	Test	2
80	O=C1NC(=O)C(=C1Sc1ccc(cc1)c1ccccc1)O	5.52	-7.5	-10.4	Training	2
81	COc1ccc2c(c1)ccc(c2)C1=C(O)C(=O)NC1=O	5.66	-7.8	-11.9	Training	2
82	CCCCCCCCCCC1=C(O)C(=O)NC1=O	7.23	-6.7	-9.1		2
83	CCCCCCCCCCCCC1=C(O)C(=O)NC1=O	7.09	-6.2	-9	Training	2
84	O=C1NC(=O)C(=C1C(=O)N)O	2.19	-5.2	-6.9	Training	2
85	O=C1NC(=O)C(=C1c1ccc(cc1)C(C)C)O	5.34	-7.2	-10.6	Test	2
86	Brc1ccc(cc1)c1csc(n1)C1=C(O)C(=O)NC1=O	6.63	-7.1	-9.3	Training	2
87	Clc1ccc(cc1)c1ccc(s1)C1=C(O)C(=O)N=C1O	6.58	-7.2	-11.1	Test	2
88	Fc1ccc(cc1)c1scc(n1)C1=C(O)C(=O)NC1=O	6.75	-7.4	-10.7	Training	2
89	Brc1ccc(cc1)c1scc(n1)C1=C(O)C(=O)NC1=O	7.00	-7.4	-9.7	Test	2
90	Brc1cccc(c1)c1scc(n1)C1=C(O)C(=O)NC1=O	6.82	-7.4	-10.2	Training	2
91	Clc1ccc(cc1)c1scc(n1)C1=C(O)C(=O)NC1=O	6.86	-7.4	-10.2	Training	2
92	O=C1NC(=O)C(=C1c1csc(n1)c1cccc(c1)C(F)(F)F)O	6.57	-7.7	-11.2	Training	2
93	Brc1ccc(cc1)Cc1scc(n1)C1=C(O)C(=O)NC1=O	6.18	-7.1	-10.6	Training	2

94	O=C1NC(=O)C(=C1c1csc(n1)c1c(Cl)cccc1Cl)O	7.07	-5.1	-9.1	Training	2
95	O=C1NC(=O)C(=C1c1csc(n1)c1cccc(c1Cl)Cl)O	6.77	-7.2	-10.3	Training	2
96	O=C1NC(=O)C(=C1c1csc(n1)c1ccc(c(c1)Cl)Cl)O	7.11	-7.4	-10.3	Training	2
97	O=C1NC(=O)C(=C1c1csc(n1)c1ccc(c(c1)Cl)C)O	6.92	-7.6	-10.6	Training	2
98	O=C1NC(=O)C(=C1c1csc(n1)c1c(C)cccc1C)O	6.92	-5.6	-9.7	Training	2
99	COc1cc(Cl)c(c(c1)Cl)c1scc(n1)C1=C(O)C(=O)NC1=O	6.77	-4.9	-9.3	Training	2
100	O=C1NC(=O)C(=C1c1csc(n1)c1ccncc1)O	6.48	-6.9	-9.8	Training	2
101	O=C1NC(=O)C(=C1c1csc(n1)c1cccnc1)O	6.16	-6.9	-9.9	Training	2
102	Cc1nc(C)cc(c1)c1scc(n1)C1=C(O)C(=O)NC1=O	6.26	-7.4	-10.1	Training	2
103	O=C1NC(=O)C(=C1c1csc(n1)c1cnccn1)O	5.33	-6.4	-9.3	Test	2
104	O=C1NC(=O)C(=C1c1csc(n1)c1cscn1)O	5.19	-6.2	-9	Training	2
105	O=C(CC(=O)C(=O)O)/C=C/C1=C(C)CCCC1(C)C	5.85	-6.9	-9.8	Test	3
106	O=C(CC(=O)C(=O)O)/C=C/c1ccccc1	4.72	-7.4	-9.5	Training	3
107	CCCCCCCCCC(=O)CC(=O)C(=O)O	5.85	-5.9	-8	Training	3
108	CCCCCCCCCc1ccc(cc1)C(=O)CC(=O)C(=O)O	6.22	-6.6	-9.9	Test	3
109	O=C(c1ccc(cc1)C1CCCCC1)CC(=O)C(=O)O	5.52	-7.8	-10.4	Training	3
110	O=C(c1ccc(cc1)c1ccccc1)CC(=O)C(=O)O	5.96	-8.2	-11.5	Training	3
111	Brc1ccc(cc1)c1ccc(cc1)C(=O)CC(=O)C(=O)O	7.20	-8.1	-10.4	Training	3
112	Sc1ccc(cc1)c1ccc(cc1)C(=O)CC(=O)C(=O)O	6.89	-7.9	-10.5	Training	3
113	CSc1ccc(cc1)c1ccc(cc1)C(=O)CC(=O)C(=O)O	6.80	-7.8	-10.5	Test	3
114	OC(=O)C(=O)CC(=O)c1ccc(cc1)c1ccc(cc1)SCc1ccccc1	6.40	-7.3	-10.4	Training	3
115	O=C(c1ccc(cc1)c1ccc(cc1)SCCc1ccncc1)CC(=O)C(=O)O	7.05	-7.4	-8.9	Test	3
116	OC(=O)C(=O)CC(=O)c1ccc(cc1)c1ccc(cc1)SCC1(O)COc2c(OC1)cccc2	7.22	-7	-6.3	Training	3
117	O=C(c1ccc(cc1)[C@H]1CCCc2c1cccc2)CC(=O)C(=O)O	5.68	-8.8	-10.6	Training	3
118	OC(=O)C(=O)CC(=O)c1ccc(cc1)Cc1ccc(c(c1)Cl)Cl	6.36	-8.5	-10.5	Training	3
119	OC(=O)C(=O)CC(=O)c1ccc(cc1)n1c(cc2c1cccc2)c1ccccc1	6.74	-7.8	1.2	Test	3
120	Clc1ccc(cc1)SC1=C(NNS1)C(=O)O	4.37	-5.5	-9	Training	4
121	Nc1ccc(c(c1)C(=O)O)O	5.11	-4.9	-6.5	Training	4
122	O=Cc1ccc(o1)c1ccc(c(c1)O)C(=O)O	4.46	-6.8	-9.6	Test	4
123	Brc1ccc2c(c1)cc(c(c2)O)C(=O)O	4.40	-6.3	-9.4	Training	4
124	ON(c1ccc(cc1)Oc1cc(C(=O)O)c(c2c1cccc2)O)O	5.54	-6.6	-5.8	Test	4
125	OC(=O)c1cc(S[C@@H]2NN=NN2c2ccccc2)c2c(c1O)cccc2	4.95	-6.6	-3.5	Training	4
126	O=Cc1ccc(o1)c1ccc(c(c1)C(=O)O)O	4.42	-7	-9.3	Training	4

127	OC(=O)c1ccc(cc1O)c1ccco1	4.41	-6.6	-9.1	Training	4
128	OCc1ccc(o1)c1ccc(c(c1)C(=O)O)O	5.34	-7.2	-9.2	Test	4
129	OCc1ccc(o1)c1ccc(c(c1)O)C(=O)O	5.10	-7.1	-9.4	Training	4
130	O=Cc1ccc(s1)c1ccc(c(c1)C(=O)O)O	5.03	-6.8	-9.1	Test	4
131	OC(=O)c1ccc(cc1O)c1cccs1	4.49	-6.5	-9.2	Test	4
132	OC(=O)c1cc(ccc1O)c1cccs1	4.40	-6.3	-8.5	Training	4
133	OC(=O)c1ccc(cc1O)c1cscc1	4.67	-6.4	-9	Training	4
134	O=N(=O)c1ccc(cc1)c1ccc(c(c1)O)C(=O)O	4.71	-7.5	-10.8	Training	4
135	COc1cccc(c1)c1ccc(c(c1)O)C(=O)O	4.71	-7.2	-10.6	Training	4
136	Oc1ccc(cc1)c1ccc(c(c1)O)C(=O)O	4.87	-7.5	-10.2	Training	4
137	OC(=O)c1ccc(cc1O)c1ccc(cc1)OCc1ccccc1	4.98	-7.8	-11.4	Training	4
138	COc1ccc(cc1)COc1ccc(cc1)c1ccc(c(c1)O)C(=O)O	4.86	-7.4	-11.6	Test	4
139	OC(=O)c1cc(ccc1O)c1ccc(cc1)OCc1ccccc1	4.83	-8.1	-11	Training	4
140	COc1ccc(cc1)COc1ccccc1c1ccc(c(c1)C(=O)O)O	5.02	-7	-8	Training	4
141	Fc1cc(OCc2ccccc2c2ccc(c(c2)O)C(=O)O)cc(c1)F	5.08	-7.6	-9.5	Training	4
142	Fc1cc(OCc2ccccc2c2ccc(c(c2)C(=O)O)O)cc(c1)F	5.24	-7.9	-8.4	Training	4
143	OC(=O)c1ccc(cc1O)c1ccccc1COc1cccc(c1)C(F)(F)F	5.36	-7.8	-9.7	Training	4
144	OC(=O)c1cc(ccc1O)c1ccccc1COc1cccc(c1)C(F)(F)F	5.46	-8.1	-9	Training	4

Table S2. R^2_{ADJ}, Q^2_{EXT}, Q^2_{CV}, number of features and MAE obtained for the best nine models

Training/Test							
**Model ID**	**Number of Features**	**R^2_{ADJ}**	**MAE**	**Q^2_{CV}-10-fold**	**MAE_{CV}-10-fold**	**Q^2_{EXT}**	**MAE_{EXT}**
**IBK1**	7	0.942	0.270	0.891	0.413	0.907	0.364
**IBK2**	3	0.925	0.308	0.865	0.436	0.863	0.391
**IBK3**	7	0.968	0.205	0.901	0.376	0.882	0.412
**IBK4**	9	0.951	0.259	0.886	0.406	0.866	0.444
**MLR1**	8	0.915	0.337	0.893	0.383	0.897	0.376
**MLR2**	11	0.915	0.349	0.894	0.389	0.912	0.348
**RF1**	7	0.987	0.134	0.889	0.382	0.907	0.310
**RF2**	10	0.987	0.138	0.987	0.376	0.864	0.366
**RF3**	13	0.986	0.136	0.879	0.389	0.888	0.361

The descriptors' abbreviations were named based on their invariant, capital letter, and physicochemical properties, which are represented in lowercase.

Table S3. Name and abbreviation of the features of the 3 selected individual models

Model	Descriptor	Abbreviation
IBK1	AC[1]_I50_B_AB_nCi_2_M1_SS0_T_KA_c-h_MID	AC1ch
IBK1	AC[1]_SD_B_AB_nCi_2_M8_MP1_D_KA_p-s_MID	ACps
IBK1	MX_B_AB_nCi_2_NS6_C_SRW_v-c_MAS	MXvc
IBK1	N1_TrF_AB_nCi_3_M25(M1)_NS0_T_LGA[1.0-2.0]_h_MID	Nh
IBK1	TIC_B_AB_nCi_2_M13_NS0_D_LGL[1-2]_c-h_MID	TIChc
IBK1	TS[1]_RA_B_AB_nCi_2_M5_NS2_D_KA_e-h_MID	TSeh
IBK1	TS[2]_RA_B_AB_nCi_2_M3_SS0_T_KA_v-c_MID	TSvc
MLR1	AM_TrF_AB_nCi_3_M25(M11)_NS0_T_LGA[1.0-2.0]_h_MID	AMh
MLR1	ES_RA_B_AB_nCi_2_M15_NS0_D_LGL[1-2]_e-c_MID	ESec
MLR1	GV[2]_SD_B_AB_nCi_2_M5_SS6_X_LGL[1-2]_e-h_MID	GVeh
MLR1	N1_B_AB_nCi_2_M13_SS0_C_LGL[2-3]_c-h_MID	Nch
MLR1	N1_B_AB_nCi_2_M16_NS0_T_LGL[1-2]_c-h_MID	N1ch
MLR1	TS[2]_RA_B_AB_nCi_2_M3_SS0_T_KA_v-c_MID	TSvc
MLR1	TS[5]_VC_F_AB_nCi_2_M3_SS0_T_KA_h_MID	TSh
MLR1	TS[6]_AM_B_AB_nCi_2_M3_NS1_T_LGL[2-3]_v-h_MID	TS6
RF1	AC[2]_RA_F_AB_nCi_2_M1_SS0_C_KA_e_MID	ACe
RF1	I50_B_AB_nCi_2_M16_SS0_A_KA_h-s_MID	Ihs
RF1	N1_B_AB_nCi_2_M1_MP0_D_LGL[2-3]_c-h_MID	Nch
RF1	N1_B_AB_nCi_2_M15_NS0_P_KA_c-h_MID	N1hc
RF1	SIC_Q_AB_nCi_2_M5_NS6_P_LGP[2]_p_MID	SICp
RF1	TS[2]_RA_B_AB_nCi_2_M3_SS0_T_KA_v-c_MID	TSvc
RF1	TS[5]_VC_B_AB_nCi_2_M8_MP0_T_KA_h-s_MID	TShs

Table S4. Correlation matrix for the MLR1's features

	AMh	TSh	TSvc	TS6	GVeh	Nch	N1ch	ESec
**AMh**	1.000	0.204	0.672	0.577	-0.528	-0.052	0.354	0.412
**TSh**	0.204	1.000	0.079	0.251	-0.121	-0.021	0.020	0.045
**TSvc**	0.672	0.079	1.000	0.501	-0.584	0.215	0.321	0.641
**TS6**	0.577	0.251	0.501	1.000	-0.401	-0.205	0.166	0.296
**GVeh**	-0.528	-0.121	-0.584	-0.401	1.000	0.111	-0.250	-0.341
**Nch**	-0.052	-0.021	0.215	-0.205	0.111	1.000	0.230	0.426
**N1ch**	0.354	0.020	0.321	0.166	-0.250	0.230	1.000	0.331
**ESec**	0.412	0.045	0.641	0.296	-0.341	0.426	0.331	1.000

Table S5. Correlation matrix for the RF1's features

	TShs	TSvc	N1hc	Ihs	ACe	Nch	SICp
**TShs**	1.000	0.135	0.243	-0.070	0.159	0.438	0.069
**TSvc**	0.135	1.000	0.241	-0.377	0.341	0.406	0.100
**N1hc**	0.243	0.241	1.000	0.026	-0.337	0.292	0.400
**Ihs**	-0.070	-0.377	0.026	1.000	-0.024	-0.335	-0.075
**ACe**	0.159	0.341	-0.337	-0.024	1.000	0.024	-0.620
**Nch**	0.438	0.406	0.292	-0.335	0.024	1.000	0.473
**SICp**	0.069	0.100	0.400	-0.075	-0.620	0.473	1.000

Table S6. Correlation matrix for the IBK1's features

	MXvc	Nh	AC1ch	TSvc	ACps	TSeh	TIChc
**MXvc**	1.000	0.435	-0.356	0.601	-0.158	-0.109	0.014
**Nh**	0.435	1.000	-0.548	0.696	-0.351	-0.046	0.330
**AC1ch**	-0.356	-0.548	1.000	-0.550	0.334	0.021	-0.272
**TSvc**	0.601	0.696	-0.550	1.000	0.005	-0.085	0.409
**ACps**	-0.158	-0.351	0.334	0.005	1.000	0.503	0.497
**TSeh**	-0.109	-0.046	0.021	-0.085	0.503	1.000	0.500
**TIChc**	0.014	0.330	-0.272	0.409	0.497	0.500	1.000

Figures

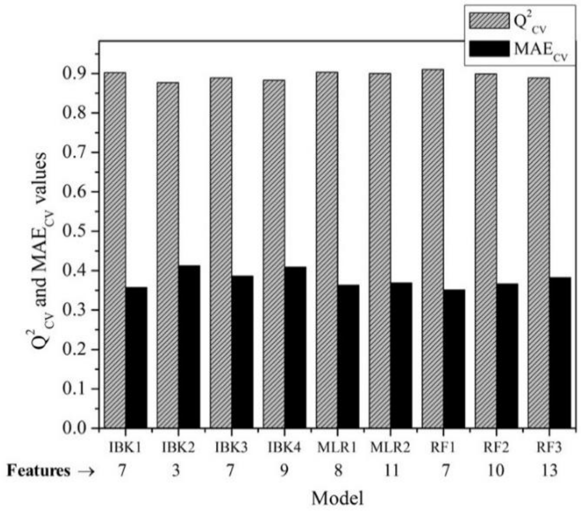

Figure S1. All dataset was considered in this analysis. Number of descriptors, 10-fold cross validation coefficients, and MAE values obtained considering the whole dataset for the 9 individual models obtained in the first screening.

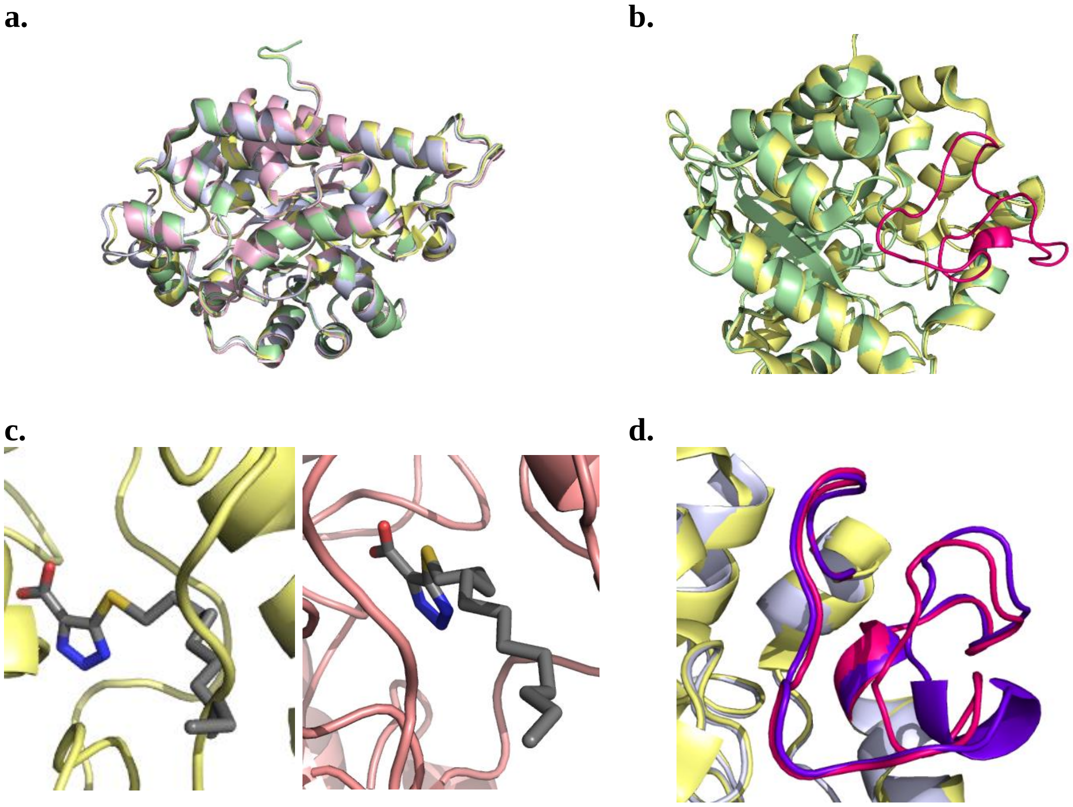

Figure S2. a. 3D structure alignment of 2RDU (yellow), 2RDT (green), 2W0U (pink), and 2RDopt (grey). b. 2RDU and 2RDT showing the missing RC (fuchsia). c. 2RDU-CDST complex before (left) and after (right) the optimization. d. RC comparison of 2RDU before (fuchsia) and after (purple) the optimization.

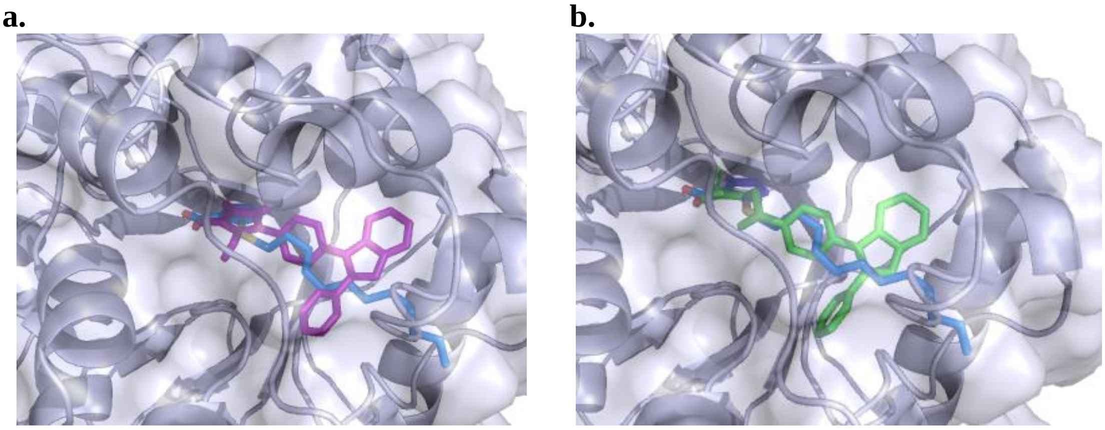

Figure S3. Docking results for compound 77 (a) and 119 (b)

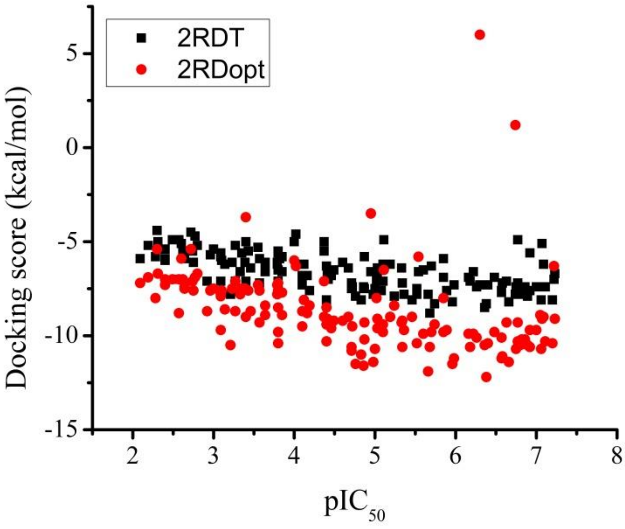

Figure S4. Experimental pIC_{50} versus docking scores.

a. Compound 73

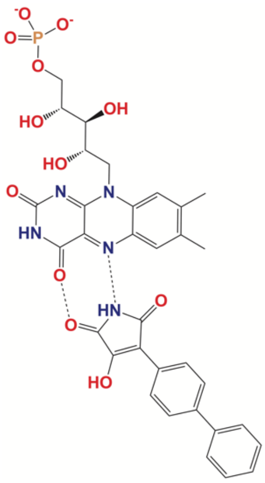

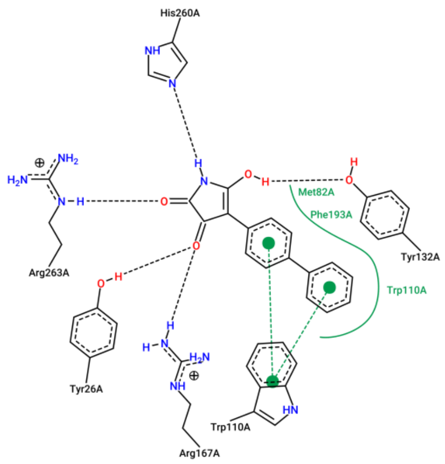

b. Compound 81

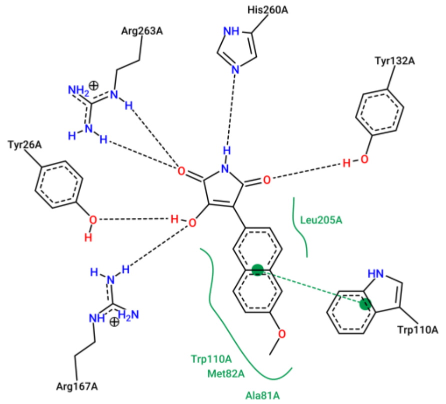

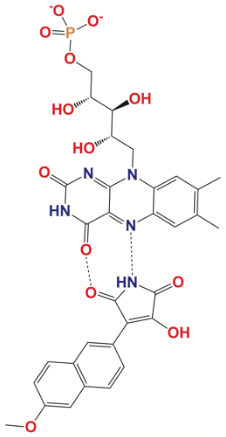

c. Compound 82

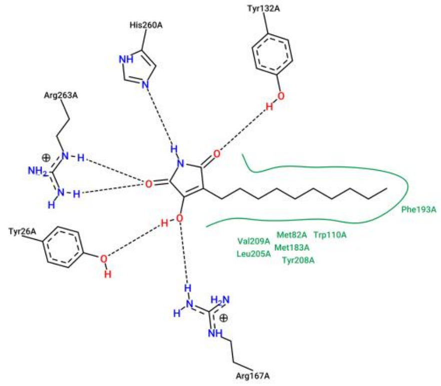

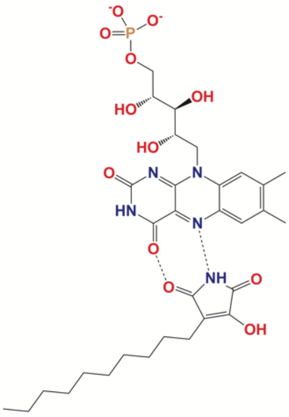

d. Compound 111

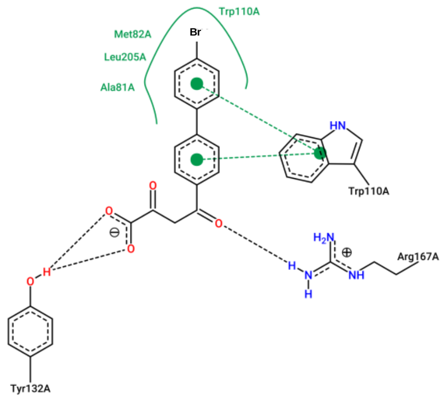

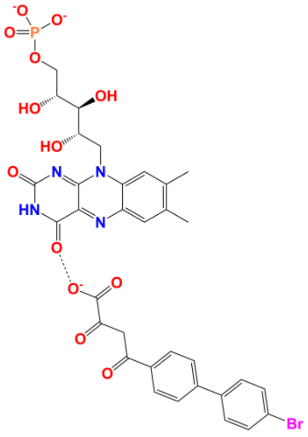

e. Compound 116

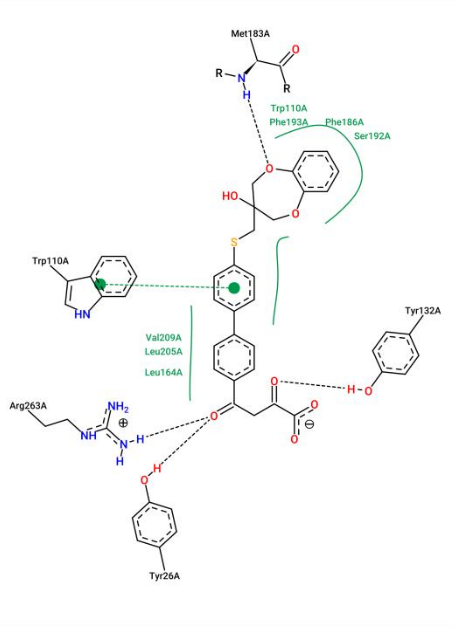

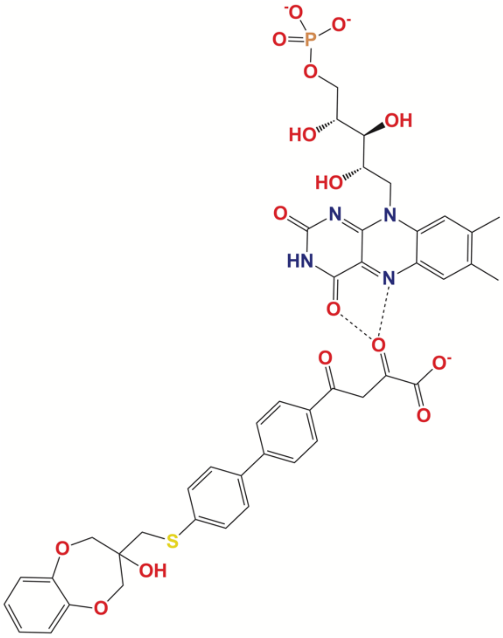

f. Compound 138

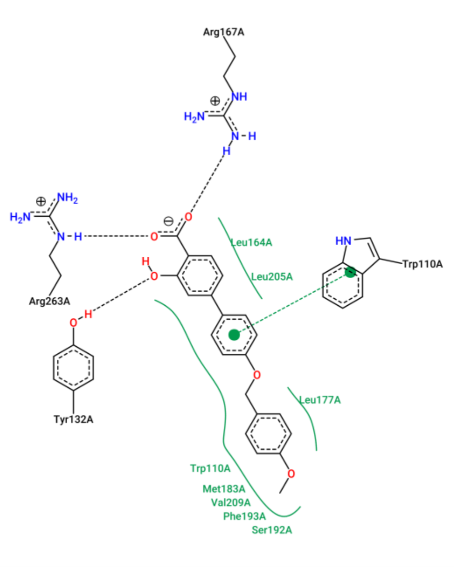

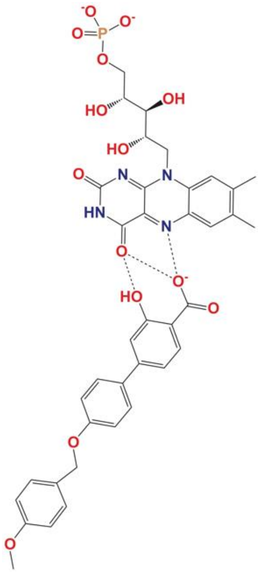

Figure S5. 2D representation of the interactions of the studied compounds with FMN and GO.

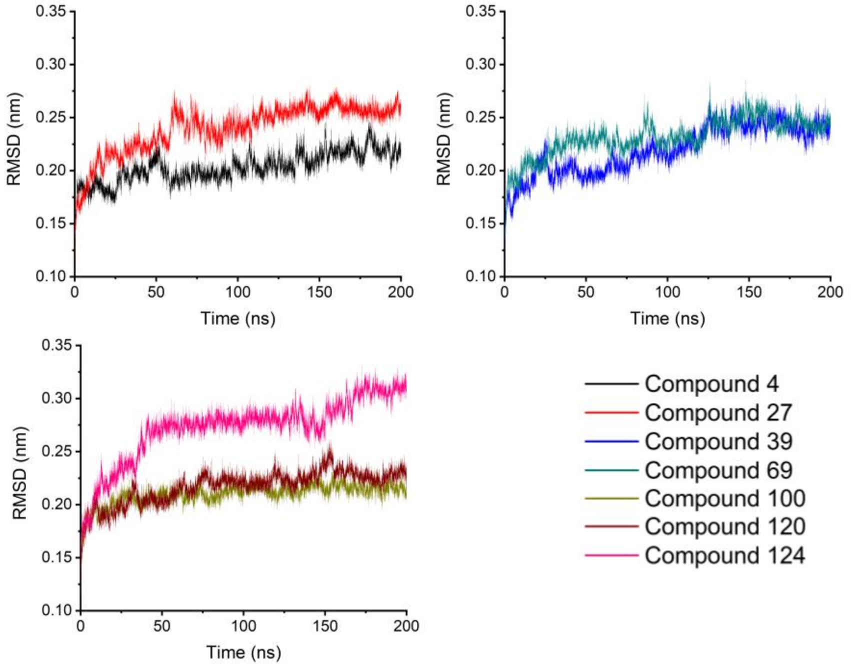

Figure S6. RMSD of GO during the 200 ns simulation.

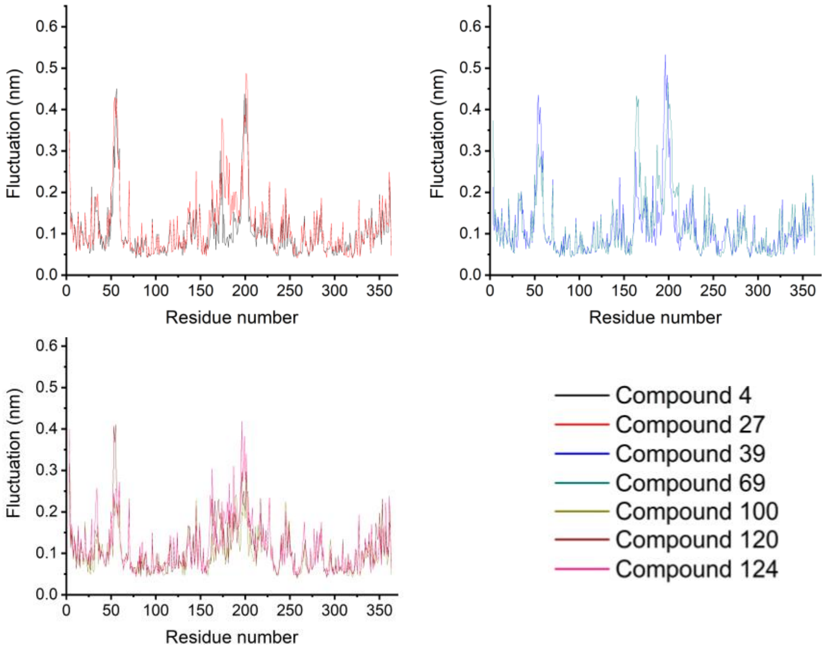

Figure S7. Root Mean Square Fluctuation (RMSF) of studied compounds.

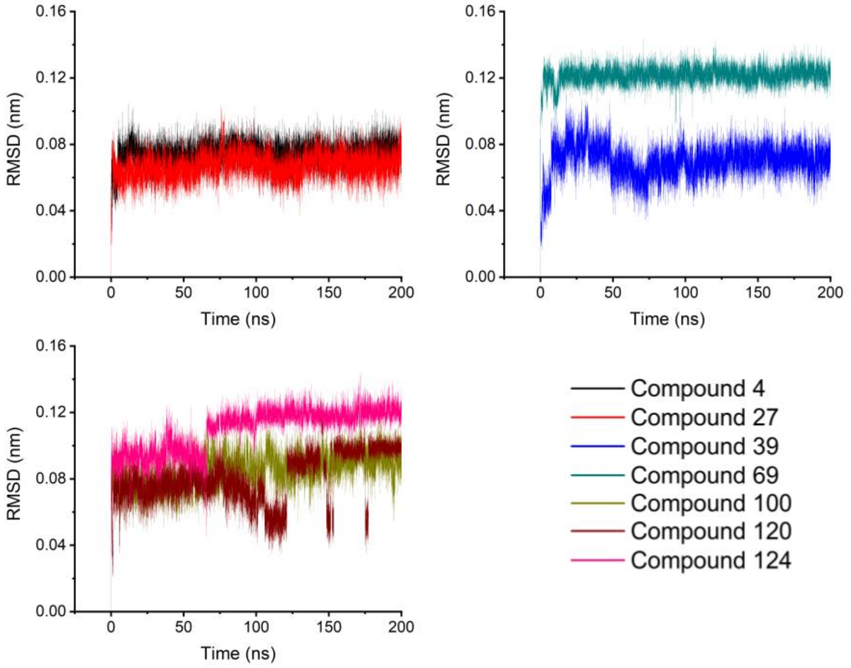

Figure S8. RMSD of FMN during the 200 ns simulation.

a.

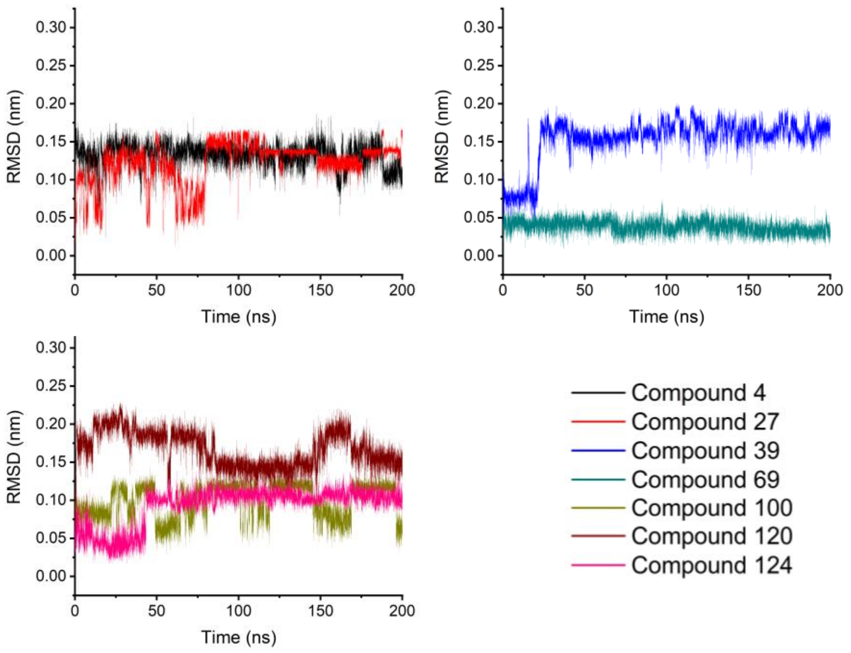

b.

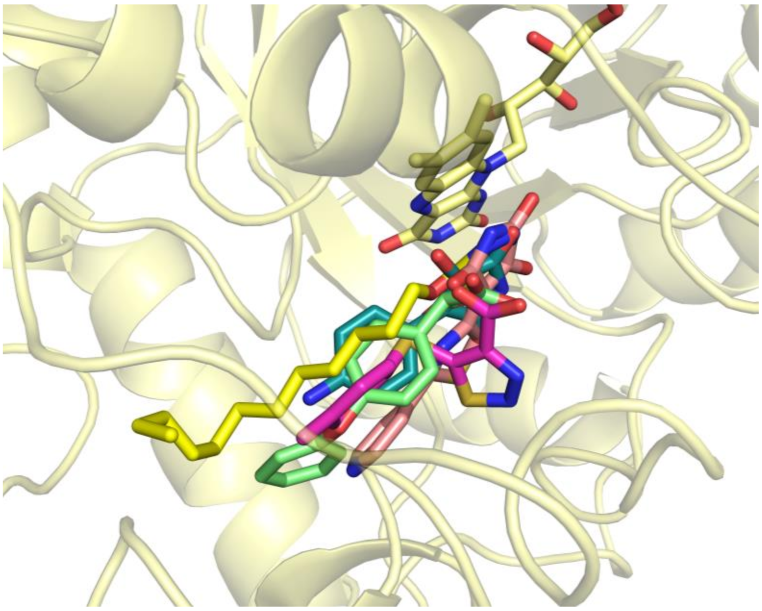

Figure S9. a. RMSD of the ligands during the 200 ns simulation. **b**. Comparison of compound 4 (light green), 69 (dark green), 100 (wheat), and 120 (pink) after 200 ns simulation vs experimental CDST (yellow).

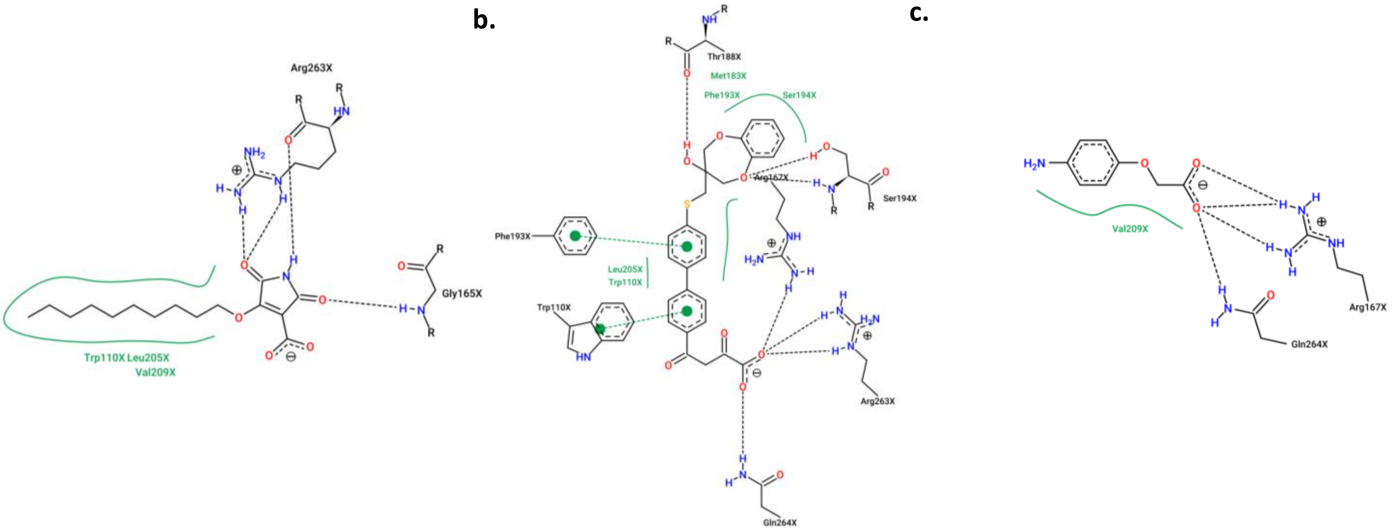

Figure S10. 2D representation of the interactions of compound 82 (a), 116 (b), and 27 (c) GO.

a.

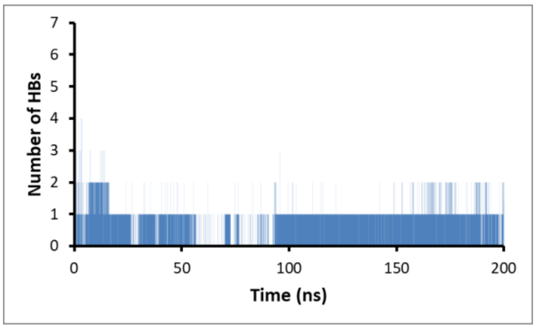
Number of HBs
0
1
2
3
4
5
6
7
0
50
100
150
200
Time (ns)

b.

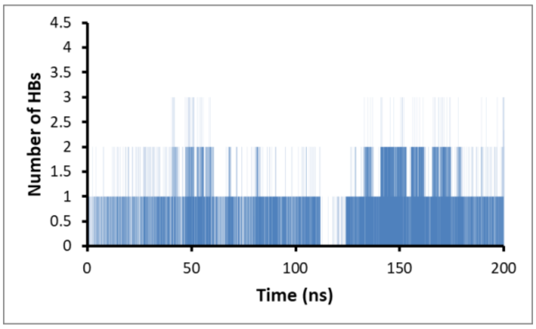
Number of HBs
0
0.5
1
1.5
2
2.5
3
3.5
4
4.5
0
50
100
150
200
Time (ns)

c.

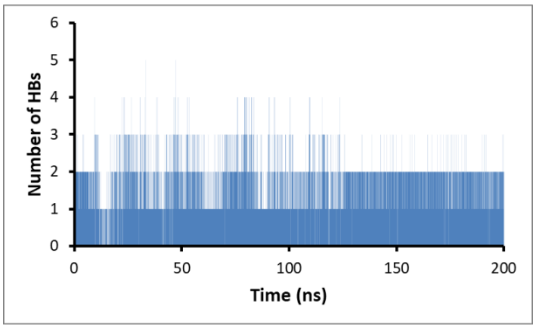
Number of HBs
0
1
2
3
4
5
6
0
50
100
150
200
Time (ns)

d.

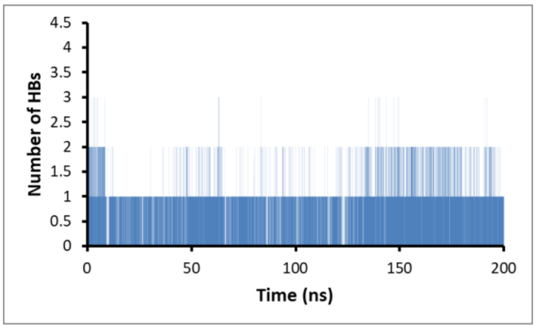
Number of HBs
0
0.5
1
1.5
2
2.5
3
3.5
4
4.5
0
50
100
150
200
Time (ns)

e.

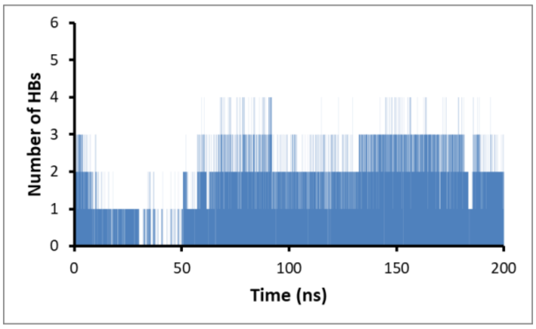
Number of HBs
0
1
2
3
4
5
6
0
50
100
150
200
Time (ns)

f.

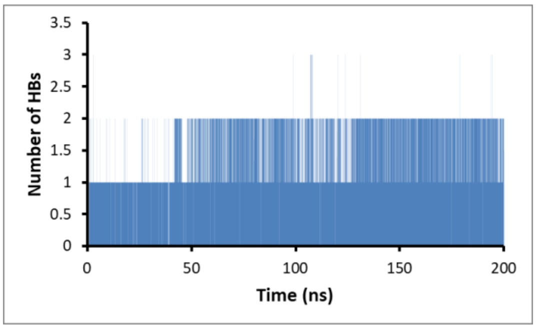
Number of HBs
0
0.5
1
1.5
2
2.5
3
3.5
0
50
100
150
200
Time (ns)

g.

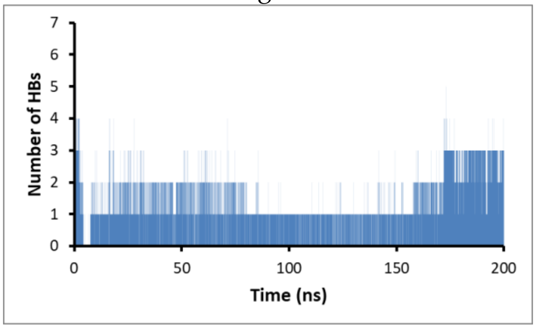
Number of HBs
0
1
2
3
4
5
6
7
0
50
100
150
200
Time (ns)

h.

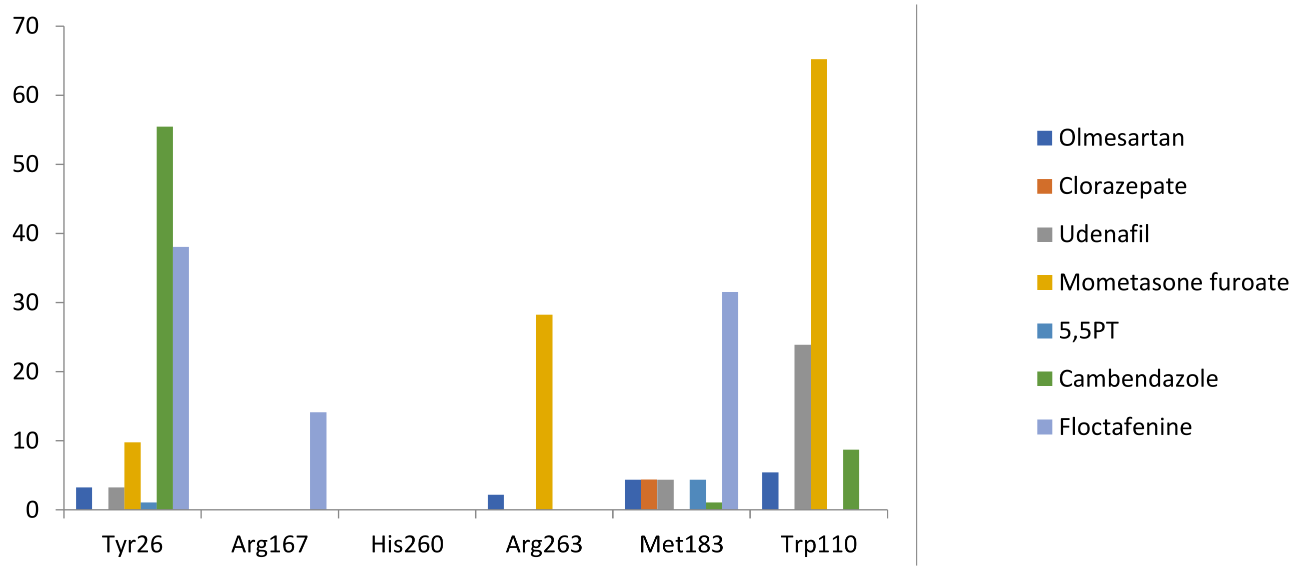

Figure S11. Number of hydrogen bonds between GO and Olmesartan (a), clorazepate (b), udenafil (c), mometasone furoate (d), 5,5PT (e), cambendazole (f), and floctafenine (g) during the 200 ns simulation and their occupancies (h).

References

1. Randall, W. C. *et al.* Quantitative structure-activity relationships involving the inhibition of glycolic acid oxidase by derivatives of glycolic and glyoxylic acids. *J. Med. Chem.* **22**, 608–614 (1979).
2. Williams, H. W. R. *et al.* Inhibitors of glycolic acid oxidase. 4-Substituted-2,4-dioxobutanoic acid derivatives. *J. Med. Chem.* **26**, 1196–1200 (1983).
3. Rooney, C. S. *et al.* Inhibitors of glycolic acid oxidase. 4-Substituted 3-hydroxy-1H-pyrrole-2,5-dione derivatives. *J. Med. Chem.* **26**, 700–714 (1983).
4. Moya-Garzón, M. D. *et al.* Salicylic Acid Derivatives Inhibit Oxalate Production in Mouse Hepatocytes with Primary Hyperoxaluria Type 1. *J. Med. Chem.* **61**, 7144–7167 (2018).
